# Management of bleeding following major trauma: a European guideline

**DOI:** 10.1186/cc5686

**Published:** 2007-02-13

**Authors:** Donat R Spahn, Vladimir Cerny, Timothy J Coats, Jacques Duranteau, Enrique Fernández-Mondéjar, Giovanni Gordini, Philip F Stahel, Beverley J Hunt, Radko Komadina, Edmund Neugebauer, Yves Ozier, Louis Riddez, Arthur Schultz, Jean-Louis Vincent, Rolf Rossaint

**Affiliations:** 1 Department of Anesthesiology, University Hospital Zurich, Rämistrasse 100, 8091 Zurich, Switzerland; 2 Charles University in Prague, Faculty of Medicine in Hradec Králové, Department of Anaesthesiology and Intensive Care Medicine, University Hospital Hradec Králové, Sokolska 581, 50005 Hradec Králové, Czech Republic; 3 Leicester Royal Infirmary, Accident and Emergency Department, Infirmary Square, Leicester LE1 5WW, UK; 4 Department of Anaesthesia and Intensive Care, University of Paris XI Faculté de Médecine Paris-Sud, 63 rue Gabriel Péri, 94276 Le Kremlin-Bicêtre, France; 5 Department of Emergency and Critical Care Medicine, University Hospital Virgen de las Nieves, ctra de Jaén s/n, 18013 Granada, Spain; 6 Department of Anaesthesia and Intensive Care, Ospedale Maggiore, Largo Nigrisoli 2, 40100 Bologna, Italy; 7 Department of Orthopaedic Surgery, Denver Health Medical Center, University of Colorado Medical School, 777 Bannock Street, Denver, CO 80204, USA; 8 Departments of Haematology, Pathology and Rheumatology, Guy's & St Thomas' Foundation Trust, Lambeth Palace Road, London SE1 7EH, UK; 9 Department of Traumatology, General and Teaching Hospital Celje, 3000 Celje, Slovenia; 10 Institute for Research in Operative Medicine, University of Witten/Herdecke, Ostmerheimerstrasse 200, 51109 Köln (Merheim), Germany; 11 Department of Anaesthesia and Intensive Care, Université René Descartes Paris 5, AP-HP, Hopital Cochin, 27 rue du Fbg Saint-Jacques, 75014 Paris, France; 12 Department of Surgery and Trauma, Karolinska University Hospital, 171 76 Solna, Sweden; 13 Ludwig-Boltzmann-Institute for Experimental and Clinical Traumatology, Donaueschingenstrasse 13, 1200 Vienna, Austria; 14 Department of Intensive Care, Erasme Hospital, University of Brussels, Belgium, route de Lennik 808, 1070 Brussels, Belgium; 15 Department of Anaesthesiology, University Hospital Aachen, Pauwelsstraße 30, 52074 Aachen, Germany

## Abstract

**Introduction:**

Evidence-based recommendations can be made with respect to many aspects of the acute management of the bleeding trauma patient, which when implemented may lead to improved patient outcomes.

**Methods:**

The multidisciplinary Task Force for Advanced Bleeding Care in Trauma was formed in 2005 with the aim of developing guidelines for the management of bleeding following severe injury. Recommendations were formulated using a nominal group process and the GRADE (Grading of Recommendations Assessment, Development, and Evaluation) hierarchy of evidence and were based on a systematic review of published literature.

**Results:**

Key recommendations include the following: The time elapsed between injury and operation should be minimised for patients in need of urgent surgical bleeding control, and patients presenting with haemorrhagic shock and an identified source of bleeding should undergo immediate surgical bleeding control unless initial resuscitation measures are successful. A damage control surgical approach is essential in the severely injured patient. Pelvic ring disruptions should be closed and stabilised, followed by appropriate angiographic embolisation or surgical bleeding control, including packing. Patients presenting with haemorrhagic shock and an unidentified source of bleeding should undergo immediate further assessment as appropriate using focused sonography, computed tomography, serum lactate, and/or base deficit measurements. This guideline also reviews appropriate physiological targets and suggested use and dosing of blood products, pharmacological agents, and coagulation factor replacement in the bleeding trauma patient.

**Conclusion:**

A multidisciplinary approach to the management of the bleeding trauma patient will help create circumstances in which optimal care can be provided. By their very nature, these guidelines reflect the current state-of-the-art and will need to be updated and revised as important new evidence becomes available.

## Introduction

Traumatic injury is the leading cause of death worldwide among persons between 5 and 44 years of age [1] and accounts for 10% of all deaths [2]. In 2002, 800,000 injury-related deaths in Europe accounted for 8.3% of total deaths [3]. Because trauma affects a disproportionate number of young people, the burden to society in terms of lost productivity, premature death, and disability is considerable. Despite improvements in trauma care, uncontrolled bleeding contributes to 30% to 40% of trauma-related deaths and is the leading cause of potentially preventable early in-hospital deaths [4-6].

Resuscitation of the trauma patient with uncontrolled bleeding requires the early identification of potential bleeding sources followed by prompt action to minimise blood loss, to restore tissue perfusion, and to achieve haemodynamic stability. Massive bleeding in trauma patients, defined here as the loss of one blood volume within 24 hours or the loss of 0.5 blood volumes within three hours, is often caused by a combination of vascular injury and coagulopathy. Contributing factors to traumatic haemorrhage include both surgical and non-surgical bleeding, prior medication, comorbidities, and acquired coagulopathy [7].

Here, we describe early diagnostic measures to identify haemorrhage that should trigger surgical or radiological interventions in most cases. Specific interventions to manage bleeding associated with pelvic ring injuries and hypothermia are discussed, as well as recommendations for the optimal application of fluid, pharmacological, blood product, and coagulation factor therapy in trauma patients.

These guidelines for the management of the bleeding trauma patient were developed by a multidisciplinary group of European experts and designated representatives from relevant professional societies to guide the clinician in the early phases of treatment. The recommendations presented here are based on a critical survey of the published literature and were formulated according to a consensus reached by the author group. Many of the critical issues faced by the treating physician have not been, and for ethical or practical reasons may never be, addressed by prospective randomised clinical studies, and therefore the formulation and grading of the recommendations presented here are weighted to reflect both this reality and the current state-of-the-art.

## Materials and methods

These recommendations were formulated and graded according the Grading of Recommendations Assessment, Development, and Evaluation (GRADE) hierarchy of evidence outlined by Guyatt and colleagues [8] and are summarised in Table 1. Comprehensive computer database literature searches were performed using the indexed online databases MEDLINE/PubMed and the Cochrane Library. Lists of cited literature within relevant articles were also screened. The primary intention of the review was to identify prospective randomised controlled trials (RCTs) and non-randomised controlled trials, existing systematic reviews, and guidelines. In the absence of such evidence, case control studies, observational studies, and case reports were considered.

**Table 1:** Grading of recommendations after Guyatt *et al*. [8]

Grade of recommendation	Clarity of risk/benefit	Quality of supporting evidence	Implications
1A			
Strong recommendation, high-quality evidence	Benefits clearly outweigh risk and burdens, or *vice versa*	Randomised controlled trials (RCTs) without important limitations or overwhelming evidence from observational studies	Strong recommendations, can apply to most patients in most circumstances without reservation
1B			
Strong recommendation, moderate-quality evidence	Benefits clearly outweigh risk and burdens, or *vice versa*	RCTs with important limitations (inconsistent results, methodological flaws, indirect, or imprecise) or exceptionally strong evidence from observational studies	Strong recommendations, can apply to most patients in most circumstances without reservation
1C			
Strong recommendation, low-quality or very low-quality evidence	Benefits clearly outweigh risk and burdens, or *vice versa*	Observational studies or case series	Strong recommendation but may change when higher-quality evidence becomes available
2A			
Weak recommendation, high-quality evidence	Benefits closely balanced with risks and burden	RCTs without important limitations or overwhelming evidence from observational studies	Weak recommendation, best action may differ depending on circumstances or patients' or societal values
2B			
Weak recommendation, moderate-quality evidence	Benefits closely balanced with risks and burden	RCTs with important limitations (inconsistent results, methodological flaws, indirect, or imprecise) or exceptionally strong evidence from observational studies	Weak recommendation, best action may differ depending on circumstances or patients' or societal values
2C			
Weak recommendation, low-quality or very low-quality evidence	Uncertainty in the estimates of benefits, risks, and burden; benefits, risk, and burden may be closely balanced	Observational studies or case series	Very weak recommendation, other alternatives may be equally reasonable

Boolean operators and Medical Subject Heading (MeSH) thesaurus keywords were applied as a standardised use of language to unify differences in terminology into single concepts. Appropriate MeSH headings and subheadings for each question were selected and modified based on search results. The scientific questions posed that led to each recommendation and the MeSH headings applied to each search are listed in Additional file 1. Searches were limited to English language abstracts and human studies; gender and age were not limited. No time-period limits were imposed on searches unless the search result exceeded 300 hits. Original publications were evaluated for abstracts that were deemed relevant. In the case of a guideline update, searches were limited to the time period following the publication of the last version of the guideline. If an acceptable systematic review or meta-analysis was identified, searches to update the data were typically limited to the time period following the search cutoff date reported in the review. Original publications were evaluated according to the levels of evidence developed by the Oxford Centre for Evidence-Based Medicine (Oxford, Oxfordshire, UK) [9].

The selection of the scientific inquiries to be addressed in the guideline, screening, and grading of the literature to be included and formulation of specific recommendations were performed by members of the Task Force for Advanced Bleeding Care in Trauma, a multidisciplinary, pan-European group of experts with specialties in surgery, anaesthesia, emergency medicine, intensive care medicine, and haematology. The core group was formed in 2004 to produce educational material on care of the bleeding trauma patient [10], on which a subsequent review article was based [11]. The Task Force consisted of the core group, additional experts in haematology and guideline development, and representatives of relevant European professional societies, including the European Shock Society, the European Society for Anaesthesia, the European Society for Emergency Medicine, the European Society for Intensive Care Medicine, and the European Trauma Society. The European Hematology Association declined the invitation to send a representative to join the Task Force. Task Force members participated in a workshop on the critical appraisal of medical literature. The nominal group process included four face-to-face meetings supplemented by several Delphi rounds [12]. The guideline development group met in June 2005 to define the scientific questions to be addressed in the guideline and again in October 2005 to finalise the scientific scope of the guidelines. Selection, screening, and grading of the literature and formulation of recommendations were accomplished in subcommittee groups consisting of at least three members via electronic or telephone communication. After distribution of the recommendations to the entire group, a further meeting of the Task Force was held in April 2006 with the aim of reaching a consensus on the draft recommendations from each subcommittee. After final refinement of specific recommendations among committee members, a subset of the Task Force met in July 2006 to finalise the manuscript document. The document was approved by the endorsing organisations in September and October 2006. An updated version of the guideline is anticipated in due time.

In the GRADE system for assessing each recommendation, the letter attached to the grade of recommendation reflects the degree of literature support for the recommendation, whereas the number indicates the level of support for the recommendation assigned by the committee of experts. Recommendations are grouped by category and somewhat chronologically in the treatment decision-making process, but not by priority or hierarchy.

## Results

### I. Initial resuscitation and prevention of further bleeding

Evidence to support the initial phase of resuscitation and prevention of further bleeding is lacking, and there have been few studies on the effect of coagulopathy on outcome. Patients with a coagulopathic condition have worse outcomes than patients of the same injury severity without a clotting disturbance [13,14], and patients with head injury also have worse outcomes in association with a coagulopathy [15]; however, contrary to popular belief, there is no evidence that patients with head injury are more likely to develop a coagulopathy than other severely injured patients [16].

There is no evidence as to whether the degree of initial bleeding affects coagulopathy. Coagulopathy is predicted by a systolic blood pressure of below 70 mm Hg [17], but this could be either a direct effect of bleeding or an associated effect of injury severity. There is no high-level scientific evidence that the initial amount of bleeding affects the patient's outcome; however, the experience of treating physicians is that uncontrolled haemorrhage is associated with poor outcome. Common experience is that wound compression prevents bleeding, but it is not known whether this reduces the incidence of coagulopathy. There is also no evidence that tells us whether control of acid-base balance during initial resuscitation affects outcome.

There is evidence to support expedient care for patients following traumatic injury; however, no study has examined the relationship between outcomes in patients transported to different types of hospital facilities and the amount of bleeding. Pre-hospital bleeding not controlled by compression and splintage requires rapid surgical or radiological intervention.

#### Recommendation 1

We recommend that the time elapsed between injury and operation be minimised for patients in need of urgent surgical bleeding control (grade 1A).

##### Rationale

Trauma patients in need of emergency surgery for ongoing haemorrhage demonstrate better survival if the elapsed time between the traumatic injury and admission to the operating theatre is minimised [18-21]. Although there are no randomised control studies to verify this statement, there are retrospective studies that provide enough evidence for early surgical intervention in these patients. This is particularly true for patients who present in an exsanguinated state or in severe haemorrhagic shock due to penetrating vascular injuries [18,19]. In accordance with these observations, Blocksom and colleagues [20] concluded that rapid resuscitation and surgical control of haemorrhage is of utmost importance and one of the prognostic determinants in a retrospective study on duodenal injuries. A retrospective study by Ertel and colleagues [21] that included 80 polytrauma patients *in extremis *or with persistent haemodynamic instability also favoured early surgical intervention to stabilise a pelvic fracture or to surgically control bleeding.

In addition, studies of different trauma systems indirectly emphasise the importance of minimising the time between initial care and surgery for those with signs of exsanguination or ongoing severe haemorrhage. Hill and colleagues [22] observed a significant decrease in mortality from shock by introducing an educational program on trauma and by establishing a 60-minute emergency department time limit for patients in a state of haemorrhagic shock. Others also stress the importance of a well-functioning system capable of timely control of haemorrhage in the exsanguinating or the severely bleeding patient [23,24]. In a retrospective review of 537 deaths in the operation room, Hoyt and colleagues [25] drew the conclusion that delayed transfer to the operating room was a cause of death that could be avoided by shortening the time required for diagnosis and resuscitation prior to surgery.

### II. Diagnosis and monitoring of bleeding

Upon patient arrival in the emergency room, an initial clinical assessment of the extent of bleeding should be employed to identify patients at risk of coagulopathy.

#### Recommendation 2

We recommend that the extent of traumatic haemorrhage be clinically assessed using a grading system such as that established by the American College of Surgeons (ACS) (grade 1C).

##### Rationale

An evaluation of the mechanism of injury (for example, blunt versus penetrating trauma) is a useful tool for determining which patients are candidates for surgical bleeding control. Table 2 summarises the four classes of physiological response and clinical signs of bleeding as defined by the ACS [26]. This type of grading system may be useful in the initial assessment of bleeding. The initial assessment can also assist in determining the next patient management goal to minimise blood loss and achieve haemodynamic stability.

**Table 2:** American College of Surgeons Advanced Trauma Life Support classification of haemorrhage severity

Haemorrhage severity according to ACS/ATLS classification^a^	Class I	Class II	Class III	Class IV
Blood loss (ml)	<750	750–1,500	1,500–2,000	>2,000
Pulse rate (per minute)	<100	>100	>120	>140
Blood pressure	Normal	Normal	Decreased	Decreased
Pulse pressure (mm Hg)	Normal	Decreased	Decreased	Decreased
Respiratory rate (per minute)	14–20	20–30	30–40	>40
Urine output (ml/hour)	>30	20–30	5–15	Negligible
Central nervous system (mental status)	Slightly anxious	Mildly anxious	Anxious, confused	Lethargic

^a^Values are estimated for a 70-kg adult. Table reprinted with permission from the American College of Surgeons [26]. ACS/ATLS, American College of Surgeons/Advanced Trauma Life Support.

#### Recommendation 3

We do not suggest hyperventilation or the use of excessive positive end-expiratory pressure (PEEP) when ventilating severely hypovolaemic trauma patients (grade 2C).

##### Rationale

There is a tendency for rescue personnel to hyperventilate patients during resuscitation [27,28], and hyperventilated trauma patients appear to have increased mortality when compared with non-hyperventilated patients [28]. The experimental correlates in animals in haemorrhagic shock may be an increased cardiac output in hypoventilated pigs [29] and a decrease in cardiac output due to 5 cm PEEP in rats [30]. In contrast, the elimination of PEEP and, to an even greater extent, negative expiratory pressure ventilation increases cardiac output and survival of rats in haemorrhagic shock [30].

#### Recommendation 4

We recommend that patients presenting with haemorrhagic shock and an identified source of bleeding undergo an immediate bleeding control procedure unless initial resuscitation measures are successful (grade 1B).

##### Rationale

The source of bleeding may be immediately obvious, and penetrating injuries are more likely to require surgical bleeding control. In a retrospective study of 106 abdominal vascular injuries, all 41 patients arriving in shock following gunshot wounds were candidates for rapid transfer to the operating theatre for surgical bleeding control [19]. A similar observation in a study of 271 patients undergoing immediate laparotomy for gunshot wounds indicates that these wounds combined with signs of severe hypovolaemic shock specifically require early surgical bleeding control. This observation is true to a lesser extent for abdominal stab wounds [31]. Data on injuries caused by penetrating metal fragments from explosives or gunshot wounds in the Vietnam War confirm the need for early surgical control when patients present in shock [18].

In blunt trauma, the mechanism of injury can determine to a certain extent whether the patient in haemorrhagic shock will be a candidate for surgical bleeding control. Only a few studies address the relationship between the mechanism of injury and the risk of bleeding, however, and none of these publications is a randomised prospective trial of high evidence. We have found no objective data describing the relationship between the risk of bleeding and the mechanism of injury of skeletal fractures in general or of long-bone fractures in particular.

Traffic accidents are the leading cause of pelvic injury. Motor vehicle crashes cause approximately 60% of pelvic fractures followed by falls from great height (23%). Most of the remainder result from motorbike collisions and vehicle-pedestrian accidents [32,33]. There is a correlation between 'unstable' pelvic fractures and intra-abdominal injuries [32,34]. An association between major pelvic fractures and severe head injuries, concomitant thoracic, abdominal, urological, and skeletal injuries is also well described [32]. High-energy injuries produce greater damage to both the pelvis and organs. Patients with high-energy injuries require more transfusion units, and more than 75% have associated head, thorax, abdominal, or genitourinary injuries [35]. It is well documented that 'unstable' pelvic fractures are associated with massive haemorrhage [34], and haemorrhage is the leading cause of death in patients with major pelvic fractures. Pelvic fractures account for 1% to 3% of all skeletal injuries. In patients with multiple trauma, the incidence of pelvic fracture increases to as much as 25% [33].

#### Recommendation 5

We recommend that patients presenting with haemorrhagic shock and an unidentified source of bleeding undergo immediate further assessment (grade 1B).

A patient in haemorrhagic shock with an unidentified source of bleeding should undergo urgent clinical assessment of chest, abdominal cavity, and pelvic ring stability using focused abdominal sonography in trauma (FAST) assessment of thorax and abdomen and/or computerised tomography (CT) examination in the shock room.

### Sonography

#### Recommendation 6

We recommend early FAST for the detection of free fluid in patients with suspected torso trauma (grade 1B).

#### Recommendation 7

We recommend that patients with significant free intra-abdominal fluid according to sonography (FAST) and haemodynamic instability undergo urgent surgery (grade 1C).

##### Rationale

Blunt abdominal trauma represents a major diagnostic challenge and an important source of internal bleeding. FAST has been established as a rapid and non-invasive diagnostic approach for detection of intra-abdominal free fluid in the emergency room [36,37]. Large prospective observational studies determined a high specificity (range 0.97 to 1.0) and a high accuracy (range 0.92 to 0.99) but low sensitivity (range 0.56 to 0.71) of initial FAST examination for detecting intra-abdominal injuries in adults and children [38-45]. Shackford and colleagues [38] assessed the accuracy of FAST performed by non-radiologist clinicians (that is, surgeons and emergency physicians who were certified for FAST by defined standards) for detecting a haemoperitoneum in 241 prospectively investigated adult patients with blunt abdominal trauma (except for *n *= 2 with penetrating injuries) during a four year period. These findings were confirmed by Richards and co-workers [39] in a four year prospective study of 3,264 adult patients with blunt abdominal trauma. Similar conclusions were drawn by the same group of investigators in a paediatric population, based on a prospective study on 744 consecutive children 16 years old or younger who underwent emergency FAST for blunt abdominal trauma [40]. Liu and colleagues [41] conducted a one year prospective comparison on the diagnostic accuracy of CT scan, diagnostic peritoneal lavage (DPL), and sonography in 55 adult patients with blunt abdominal trauma. The authors found a high sensitivity (0.92), specificity (0.95), and accuracy (0.93) of initial FAST examination for the detection of haemoperitoneum. Although CT scan and DPL were shown to be more sensitive (1.0 for DPL, 0.97 for CT) than sonography for detection of haemoperitoneum, these diagnostic modalities are more time-consuming (CT and DPL) and invasive (DPL) [41].

The hypotensive patient (systolic blood pressure below 90 mm Hg) presenting free intra-abdominal fluid according to FAST is a potential candidate for early surgery if he or she cannot be stabilised by initiated fluid resuscitation, according to a retrospective study of 138 patients by Farahmand and colleagues [46]. A similar conclusion can be drawn from a prospective blinded study of 400 hypotensive blunt trauma victims (systolic blood pressure below 90 mm Hg) showing that specific levels of intra-abdominal fluid detected by FAST in these patients was an accurate indicator of the need for urgent surgery [47]. In addition, a retrospective study by Rozycki and colleagues [48] of 1,540 patients (1,227 blunt, 313 penetrating trauma) assessed with FAST as an early diagnostic tool showed that the ultrasound examination had a sensitivity and specificity close to 100% when the patients were hypotensive.

A number of patients who present free intra-abdominal fluid according to FAST can safely undergo further investigation with multi-slice spiral computed tomography (MSCT). Under normal circumstances, adult patients need to be haemodynamically stable when MSCT is performed outside of the emergency room. In the retrospective study of 1,540 patients (1,227 blunt, 313 penetrating trauma) who were assessed early with FAST, a successful non-operative management was achieved in 24 (48%) of the 50 patients who were normotensive on admission and had true positive sonographic examinations. These results justified an MSCT scan of the abdomen rather than an immediate exploratory laparotomy [48]. In a review article, Lindner and colleagues [49] also concluded that the haemodynamically stable patient should undergo MSCT scanning regardless of the findings from ultrasound or clinical examination.

### Computer tomography

#### Recommendation 8

We recommend that haemodynamically stable patients with suspected head, chest, and/or abdominal bleeding following high-energy injuries undergo further assessment using CT (grade 1C).

##### Rationale

The increasing role of MSCT in the imaging concept of acute trauma patients is well documented [50-55]. The integration of modern MSCT scanners in the emergency room area allows the immediate examination of trauma victims following admission [52,53].

Using modern 16-slice CT scanners, total whole-body scanning time amounts to approximately 120 seconds. Sixty-four-slice CT scanners may reduce scanning time to less than 30 seconds. In a retrospective study comparing 370 patients in two groups, Weninger and colleagues [53] showed that the full extent of injury was definitively diagnosed 12 ± 9 minutes following application of the MSCT protocol. In the group of conventionally diagnosed patients, definitive diagnosis was possible after 41 ± 27 minutes. Faster diagnosis led to shorter emergency room and operating room time and shorter intensive care unit (ICU) stay [53]. Compared to MSCT, all traditional techniques of diagnostic and imaging evaluation have some limitations. The diagnostic accuracy, safety, and effectiveness of immediate MSCT is dependent on sophisticated pre-hospital treatment by trained and experienced emergency personnel and short transportation times [56,57].

If an MSCT is not available in the emergency room, the realisation of CT scanning implies transportation of the patient to the CT room, and therefore the clinician must evaluate the implications and potential risks and benefits of the procedure. According to established standards, such as those developed by the ACS, only the haemodynamically stable patient should be considered for CT scanning. During transport to the MSCT and imaging, all vital signs should be closely monitored and resuscitation measures continued.

For those patients in whom haemodynamic stability is questionable, imaging techniques such as ultrasound and chest and pelvic radiography may be useful. Peritoneal lavage is rarely indicated if ultrasound or CT is available [58]. Transfer times to and from all forms of diagnostic imaging need to be considered carefully in any patient who is haemodynamically unstable. In addition to the initial clinical assessment, near-patient testing results, including full blood count, haematocrit (Hct), blood gases, and lactate, should be readily available under ideal circumstances.

### Haematocrit

#### Recommendation 9

We do not recommend the use of single Hct measurements as an isolated laboratory marker for bleeding (grade 1B).

##### Rationale

Hct measurements are part of the basic diagnostic work-up for trauma patients. The diagnostic value of the Hct for detecting trauma patients with severe injury and occult bleeding sources has been a topic of debate in the past decade [59-61]. A major limit of the diagnostic value is the confounding influence of resuscitative measures on the Hct due to administration of intravenous fluids and red cell concentrates [61-64]. A retrospective study of 524 trauma patients determined a low sensitivity (0.5) of the initial Hct on admission for detecting those patients with an extent of traumatic haemorrhage requiring surgical intervention [61].

Two prospective observational diagnostic studies determined the sensitivity of serial Hct measurements for detecting patients with severe injury [59,60]. Paradis and colleagues [59] found that the mean change in Hct between arrival and 15 minutes and between 15 and 30 minutes was not significantly different between patients with serious injuries (*n *= 21) compared to trauma patients without serious injuries (*n *= 39). Whereas a decrease in Hct of more than or equal to 6.5% at 15 and 30 minutes had a high specificity (0.93 to 1.0) for a serious injury, the sensitivity for detecting severely injured patients was very low (0.13 to 0.16) [59]. The authors also found that a normal Hct on admission did not preclude a significant injury [59]. Zehtabchi and colleagues [60] expanded the time window of serial Hct assessments to fourhours after arrival. All trauma patients requiring a blood transfusion within the first fourhours were excluded from the study. In the remaining 494 patients, a decrease in Hct of more than 10% between admission and fou hours was highly specific (0.92 to 0.96) for severe injury but was associated with a very low sensitivity (0.09 to 0.27) for detecting patients with significant injuries [60]. The limitation of the high specificity of the decrease in Hct after fourhours in this study is that it included only trauma patients who did not receive any blood transfusions during the first fourhours [60]. In summary, decreasing serial Hct measurements may reflect continued bleeding, but the patient with significant bleeding may maintain his or her serial Hct.

### Serum lactate

#### Recommendation 10

We recommend serum lactate measurement as a sensitive test to estimate and monitor the extent of bleeding and shock (grade 1B).

##### Rationale

Serum lactate has been used as a diagnostic parameter and prognostic marker of haemorrhagic shock since the 1960s [65]. The amount of lactate produced by anaerobic glycolysis is an indirect marker of oxygen debt, tissue hypoperfusion, and the severity of haemorrhagic shock [66-69]. Vincent and colleagues [70] reported on the value of serial lactate measurements in predicting survival in a prospective study on a heterogenic group of 27 patients with circulatory shock. The authors concluded that changes in lactate concentrations provide an early and objective evaluation of a patient's response to therapy and suggested that repeated lactate determinations represent a reliable prognostic index for patients with circulatory shock [70]. Abramson and colleagues [71] performed a prospective observational study on patients with multiple trauma to evaluate the correlation between lactate clearance and survival. Patients who died within the first 48 hours (*n *= 25) were excluded from the study. The remaining 76 patients were analysed with respect to the time of serum lactate normalisation compared between survivors and non-survivors who died after 48 hours [71]. Survival was 100% in those patients in whom lactate levels returned to the normal range (≤ 2 mmol/l) within 24 hours. Survival decreased to 77.8% if normalisation occurred within 48 hours and to 13.6% in those patients in whom lactate levels were elevated above 2 mmol/l for more than 48 hours [71]. These findings were confirmed in a study on 129 trauma patients by Manikis and colleagues [72]. The authors found that the initial lactate levels were higher in non-survivors than in survivors and that the prolonged time for normalisation of lactate levels of more than 24 hours was associated with the development of post-traumatic organ failure [72]. Together, both the initial serum lactate and serial lactate levels are reliable indicators of morbidity and mortality following trauma [71,72].

### Base deficit

#### Recommendation 11

We recommend base deficit as a sensitive test to estimate and monitor the extent of bleeding and shock (grade 1C).

##### Rationale

Base deficit values derived from arterial blood gas analysis provide an indirect estimation of global tissue acidosis due to impaired perfusion [66,68]. Siegel [73] demonstrated that the initial base deficit represented an independent single predictor of post-traumatic mortality in 185 patients with blunt liver trauma. Two large retrospective studies on 3,791 [74] and 2,954 [75] trauma patients have strengthened the utility of the initial base deficit as a sensitive diagnostic marker of the degree and duration of inadequate perfusion and as a prognostic parameter for post-traumatic complications and death. Davis and colleagues [75] stratified the extent of base deficit into three categories: mild (-3 to -5 mEq/l), moderate (-6 to -9 mEq/l), and severe (less than -10 mEq/l). Based on this stratification, they established a significant correlation between the admission base deficit and transfusion requirements within the first 24 hours and the risk of post-traumatic organ failure or death [75]. In a different retrospective study, the same group of authors showed that the base deficit is a better prognostic marker of death than the pH in arterial blood gas analyses [76]. Furthermore, the base deficit was shown to represent a highly sensitive marker for the severity of injury and the incidence of post-traumatic death, particularly in trauma patients older than 55 years of age [77]. In paediatric patients, admission base deficit was also shown to correlate significantly with the extent of post-traumatic shock and mortality, as determined in a retrospective study which included 65 critically injured children and used a cutoff value of less than -5 mEq/l [78]. However, in contrast to the data on lactate levels in haemorrhagic shock, reliable large-scale prospective studies on the correlation between base deficit and outcome are still lacking.

Although both the base deficit and serum lactate levels are well correlated with shock and resuscitation, these two parameters do not strictly correlate with each other in severely injured patients [79]. Therefore, the independent assessment of both parameters is recommended for the evaluation of shock in trauma patients [66,68,79,80]. Composite scores that assess the likelihood of massive transfusion and that include base deficit and other clinical parameters have been developed but require further validation [80,81].

### III. Rapid control of bleeding

#### Recommendation 12

We recommend that patients with pelvic ring disruption in haemorrhagic shock undergo immediate pelvic ring closure and stabilisation (grade 1B).

#### Recommendation 13

We recommend that patients with ongoing haemodynamic instability despite adequate pelvic ring stabilisation receive early angiographic embolisation or surgical bleeding control, including packing (grade 1B).

##### Rationale

Markers of pelvic haemorrhage include anterior-posterior and vertical shear deformations, CT 'blush' (active arterial extravasation), bladder compression pressure, pelvic haematoma volumes greater than 500 ml evident by CT, and ongoing haemodynamic instability despite adequate fracture stabilisation [82-85]. Initial therapy of pelvic fractures includes control of venous and/or canellous bone bleeding by pelvic closure [86]. Some institutions use primarily external fixators to control haemorrhage from pelvic fractures [82], but pelvic closure may also be achieved using a bed sheet, pelvic binder, or a pelvic C-clamp [86-90]. Although arterial haemorrhage from pelvic fractures may be lethal, venous bleeding may be equally devastating. Arterial embolisation appears to achieve its effect by controlling the arterial bleeding and allowing the tamponade effect of the haematoma to control venous bleeding [91,92].

Results of surgery to control pelvic haemorrhage via laparotomy have remained poor due to the existence of an extensive collateral circulation. However, in suboptimal situations (for example, when embolisation is not possible), extraperitoneal packing of the pelvis may reduce the loss of blood. Extraperitoneal haemorrhage in patients with haemorrhagic shock and pelvic ring disruption may be attributed to ruptured veins, fracture surfaces, and/or arterial sources. The overall mortality rate of patients with severe pelvic ring disruptions and haemodynamic instability remains as high as 30% to 45% [93]. Angioembolisation is often applied in patients with ongoing haemodynamic instability despite adequate fracture stabilisation and the exclusion of extra-pelvic sources of haemorrhage. Repeat angiography may be of value in those selected patients [86]. Patients who require embolisation tend to be older, have a higher injury severity score, and are more likely to be coagulopathic and haemodynamically unstable than patients who not require embolisation [94].

#### Recommendation 14

We recommend that early bleeding control be achieved by packing, direct surgical bleeding control, and the use of local haemostatic procedures. In the exsanguinating patient, aortic cross-clamping may be employed as an adjunct to achieve bleeding control (grade 1C).

##### Rationale

The choice of thoracic or abdominal aortic clamping should be determined according to the site of bleeding, available surgical skill, and speed. The patient in haemorrhagic shock in whom immediate aortic cross-clamping is warranted is characterised by an injury to the torso and the severity of the blood loss and shock. The hypotensive state will not respond to the intravenous resuscitation and may lead to cardiac arrest. The cause of injury is predominantly penetrating (for example, a gunshot wound or a stab wound). Depending on the cause of injury, the mortality rate in these situations is extremely high [18,19,95]. However, when the source of bleeding is intra-abdominal, thoracic aortic clamping combined with other measures for haemorrhage control can be life-salvaging in nearly one third of patients, according to Millikan and Moore [96] and Cothren and Moore [97]. It is unclear whether the thoracic aortic clamping should be performed before or after the abdominal incision [98]. No study has compared thoracic aortic clamping above the diaphragm with abdominal aortic clamping just below the diaphragm, although the latter method is favoured by some surgeons [98].

The cross-clamping of the aorta should be considered as an adjunct to other initial haemorrhage control measures such as the evacuation of blood, direct surgical bleeding control, or packing of bleeding sources [99]. When aortic clamping is deemed necessary due to continuous bleeding or low blood pressure, the prognosis is generally poor [100].

#### Recommendation 15

We recommend that damage control surgery be employed in the severely injured patient presenting with deep haemorrhagic shock, signs of ongoing bleeding, and coagulopathy. Additional factors that should trigger a damage control approach are hypothermia, acidosis, inaccessible major anatomic injury, a need for time-consuming procedures, or concomitant major injury outside the abdomen (grade 1C).

##### Rationale

The severely injured patient arriving to the hospital with continuous bleeding or deep haemorrhagic shock generally has a poor chance of survival unless early control of bleeding, proper resuscitation, and blood transfusion are achieved. This is particularly true for patients who present with uncontrolled bleeding due to multiple penetrating injuries as well as patients with multiple injuries and unstable pelvic fractures with ongoing bleeding from fracture sites and retroperitoneal vessels. The common denominator in these patients is the exhaustion of physiological reserves with resulting profound acidosis, hypothermia, and coagulopathy. In the trauma community, this is also called the 'bloody vicious cycle' or the 'lethal triad.' In 1983, Stone and colleagues [101] described the techniques of abbreviated laparotomy, packing to control haemorrhage and of deferred definitive surgical repair until coagulation had been established. Since then, a number of authors have described the beneficial results of this concept, which is now called 'damage control' [31,33,87,90,101-104]. Damage control consists of three components. The first component is an abbreviated resuscitative laparotomy for control of bleeding, the restitution of blood flow where necessary, and the control of contamination. This should be achieved as quickly as possible without spending unnecessary time on traditional organ repairs that can be deferred to a later phase. The abdomen is packed and temporary abdominal closure is performed. The second component is intensive care treatment, focused on core rewarming, correction of the acid-base imbalance, and coagulopathy as well as optimising the ventilation and the haemodynamic status. Further diagnostic investigations are also frequently performed during this phase. The third component is the definitive surgical repair that is performed only when target parameters have been achieved [99,105-107].

Despite the lack of controlled randomised studies comparing damage control to traditional surgical management, a retrospective review by Stone and colleagues [101] presents data in favour of damage control for the severely injured patient presenting signs of coagulopathy during surgery. Rotondo and colleagues [102] found similar results in a subgroup of patients with major vascular injury and two or more visceral injuries, and Carrillo and colleagues [103] demonstrated the benefit of damage control in patients with iliac vessel injury. In addition, a cumulative review of 961 patients treated with damage control reported overall mortality and morbidity rates of 52% and 40%, respectively [106].

### IV. Tissue oxygenation, type of fluid, and hypothermia

#### Recommendation 16

We suggest a target systolic blood pressure of 80 to 100 mm Hg until major bleeding has been stopped in the initial phase following trauma without brain injury (grade 2C).

##### Rationale

To maintain tissue oxygenation, traditional treatment of trauma patients uses early and aggressive fluid administration to restore blood volume. However, this approach may increase the hydrostatic pressure on the wound and cause a dislodgement of blood clots, a dilution of coagulation factors, and undesirable cooling of the patient. The concept of low-volume fluid resuscitation, so-called 'permissive hypotension,' avoids the adverse effects of early aggressive resuscitation while maintaining a level of tissue perfusion that, although lower than normal, is adequate for short periods [108]. Its general effectiveness remains to be confirmed in randomised clinical trials, but studies have demonstrated increased survival when a low-volume fluid resuscitation concept was used in penetrating trauma [109,110]. In contrast, no significant difference was found in patients with blunt trauma [111]. One study concluded that mortality was higher after on-site resuscitation compared with in-hospital resuscitation [112]. It seems that greater increases in blood pressure are tolerated without exacerbating haemorrhage when they are achieved gradually and with a significant delay following the initial injury [113]. All the same, a recent Cochrane systematic review concluded that there is no evidence from randomised clinical trials for or against early or larger volumes of intravenous fluids in uncontrolled haemorrhage [114]. The low-volume approach is contraindicated in traumatic brain injury and spinal injuries because an adequate perfusion pressure is crucial to ensure tissue oxygenation of the injured central nervous system. In addition, the concept of permissive hypotension should be considered carefully in the elderly patient and may be contraindicated if the patient suffers from chronic arterial hypertension.

Red blood cell (RBC) transfusion enables the maintenance of oxygen transport in some patients. Early signs of inadequate circulation are relative tachycardia, relative hypotension, oxygen extraction greater than 50%, and PvO_2_(mixed venous oxygen pressure) of less than 32 mm Hg [115-117]. The depth of shock, haemdoynamic response to resuscitation, and the rate of actual blood loss in the acutely bleeding and haemodynamically unstable patient may also be integrated into the indication for RBC transfusion. In general, RBC transfusion is recommended to maintain haemoglobin (Hb) between 7 and 9 g/dl [118].

#### Recommendation 17

We suggest that crystalloids be applied initially to treat the bleeding trauma patient. Colloids may be added within the prescribed limits for each solution (grade 2C).

##### Rationale

It is still unclear which type of fluid should be employed in the initial treatment of the bleeding trauma patient. Although several meta-analyses have shown an increased risk of death in patients treated with colloids compared with patients treated with crystalloids [119-123] and three of these studies showed that the effect was particularly significant in a trauma subgroup [119,122,123], a more recent meta-analysis showed no difference in mortality between colloids and crystalloids [124]. Problems in evaluating and comparing the use of different resuscitation fluids include the heterogeneity of populations and therapy strategies, limited quality of analysed studies, mortality not always being the primary outcome, and different (often short) observation periods. It is therefore difficult to reach a definitive conclusion as to the advantage of one type of resuscitation fluid over the other. The SAFE (Saline versus Albumin Fluid Evaluation) study compared 4% albumin with 0.9% sodium chloride in 6,997 ICU patients and showed that albumin administration was not associated with worse outcomes; however, there was a trend toward higher mortality in the trauma subgroup that received albumin (*p *= 0.06) [125]. Promising results have been obtained with hypertonic solutions. One study showed that use of hypertonic saline was associated with lower intracranial pressure than with normal saline in brain-injured patients [126], and a meta-analysis comparing hypertonic saline dextran with normal saline for resuscitation in hypotension from penetrating torso injuries showed improved survival in the hypertonic saline dextran group when surgery was required [127]. A clinical trial with brain injury patients found that hypertonic saline reduced intracranial pressure more effectively than dextran solution with 20% mannitol [128]. However, Cooper and colleagues [129] found almost no difference in neurological function six months after traumatic brain injury in patients who had received pre-hospital hypertonic saline resuscitation compared to conventional fluid.

#### Recommendation 18

We recommend early application of measures to reduce heat loss and warm the hypothermic patient in order to achieve and maintain normothermia (grade 1C).

##### Rationale

Hypothermia, defined as a core body temperature of less than 35°C, is associated with acidosis, hypotension, and coagulopathy in severely injured patients. In a retrospective study with 122 patients, hypothermia was an ominous clinical sign, accompanied by high mortality and blood loss [130]. The profound clinical effects of hypothermia ultimately lead to higher morbidity and mortality, and hypothermic patients require more blood products [131].

Hypothermia is associated with an increased risk of severe bleeding, and hypothermia in trauma patients represents an independent risk factor for bleeding and death [132]. The effects of hypothermia include altered platelet function, impaired coagulation factor function (a 1°C decrease in temperature is associated with a 10% decrease in function), enzyme inhibition, and fibrinolysis [133,134]. Body temperatures below 34°C compromise blood coagulation, but this has been observed only when coagulation tests, prothrombin time [PT] and activated partial thromboplastin time [aPTT] are carried out at the low temperatures observed in patients with hypothermia and not when assessed at 37°C, the temperature typically used for such tests. Steps to prevent hypothermia and the risk of hypothermia-induced coagulopathy include removing wet clothing, covering the patient to avoid additional heat loss, increasing the ambient temperature, forced air warming, warm fluid therapy, and (in extreme cases) extracorporeal re-warming devices [135,136].

Animal and human studies of controlled hypothermia in haemorrhage have shown some positive results compared with normothermia [137,138]. In 2003, McIntyre and colleagues [139] published a meta-analysis showing a beneficial effect on mortality rates and neurological outcome when using mild hypothermia in traumatic brain injury. In contrast, in 2004, one meta-analysis analysed the effect of hypothermia in traumatic brain injury using the results of eight studies with predefined criteria for RCTs; no reduction in mortality rates and only a slight benefit in neurological outcome could be demonstrated [140]. These contradictory results may be due to the different exclusion and inclusion criteria for the studies used for the analysis. Henderson and colleagues [140] included two studies in which patients without increased intracranial pressure were enrolled. Had these two studies been excluded from the meta-analysis, a benefit with respect to improved neurological outcome might have been demonstrated [141]. Moreover, the studies included differed with respect to the speed of induction and duration of hypothermia, which may be very important factors influencing the benefit of this treatment.

If mild hypothermia is applied in traumatic brain injury, cooling should take place within the first 3 hours following injury and be maintained for approximately 48 hours, rewarming should last 24 hours, and the cerebral perfusion pressure should be maintained above 50 mm Hg (70 mm Hg). Patients most likely to benefit from hypothermia are those with a Glasgow Coma Scale of between 4 and 7 at admission [142]. Possible side effects are hypotension, hypovolaemia, electrolyte disorders, insulin resistance, reduced insulin secretion, and increased risk of infection [143]. Further studies are warranted to investigate the postulated benefit of hypothermia in traumatic brain injury, taking these important factors into account.

### V. Management of bleeding and coagulation

#### RBCs, fresh frozen plasma, and platelets

#### Recommendation 19

We recommend a target Hb of 7 to 9 g/dl (grade 1C).

##### Rationale

There is experimental evidence that erythrocytes are involved in the biochemical and functional responsiveness of activated platelets, suggesting that erythrocytes contribute to haemostasis. In addition to the rheological effect on the margination of platelets, red cells support thrombin generation [144]. However, the optimal Hct or Hb concentration required to sustain haemostasis in massively bleeding patients is unclear. Further investigations into the role of the Hb concentration on haemostasis in massively transfused patients are therefore warranted.

The specific effect of the Hct on blood coagulation is largely unknown [145]. An acute reduction of the Hct may result in an increase in the bleeding time [146,147] with restoration upon re-transfusion [146]. This may be related to the presence of the enzyme elastase on the surface of RBC membranes, which may activate coagulation factor IX, thereby triggering blood coagulation [148,149]. However, a moderate reduction of the Hct does not increase blood loss from a standard spleen injury [147], and an isolated *in vitro *reduction of the Hct did not compromise blood coagulation as assessed by thromboelastography [150].

No prospective randomised trial has compared restrictive and liberal transfusion regimens in trauma, but 203 trauma patients from the Transfusion Requirements in Critical Care (TRICC) trial [151] were re-analysed [118]. A restrictive transfusion regimen (Hb transfusion trigger less than 7.0 g/dl) resulted in fewer transfusions as compared with the liberal transfusion regimen (Hb transfusion trigger less than 10 g/dl) and appeared to be safe. However, no statistically significant benefit in terms of multiple organ failure or post-traumatic infections was observed. It should be emphasised that this study was neither designed nor powered to answer these questions with precision. In addition, it cannot be ruled out that the number of RBC units transfused reflects merely the severity of injury. Therefore, the observed correlation between numbers of RBC units transfused and multiple organ failure [152] may reflect a correlation between the severity of injury and multiple organ failure. Adequately powered studies similar to the TRICC trial are therefore urgently needed in post-traumatic patients.

Despite the lack of high-level scientific evidence for a specific Hb transfusion trigger in patients with traumatic brain injury, these patients are currently transfused in many centres to achieve an Hb of approximately 10 g/dl [153]. This may be justified by the recent finding that increasing the Hb from 8.7 to 10.2 g/dl improved local cerebral oxygenation [154]. It remains unclear, however, whether this practice will result in an improved neurological outcome. Although the lowest Hct was correlated with adverse neurological outcome, RBC transfusions were equally found to be an independent factor for adverse neurological outcome in a recent retrospective study [155]. Interestingly, the number of days with an Hct below 30% was found to be correlated with an improved neurological outcome. Therefore, the authors suggest that patients with severe traumatic brain injury should not have an Hb transfusion threshold different than that of other critically ill patients [155].

#### Recommendation 20

We recommend treatment with thawed fresh frozen plasma (FFP) in patients with massive bleeding or significant bleeding complicated by coagulopathy (PT or aPTT more than 1.5 times control). The initial recommended dose is 10 to 15 ml/kg, but further doses may be required (grade 1C).

##### Rationale

The clinical efficacy of FFP is largely unproven [156]. Nevertheless, most guidelines recommend the use of FFP either in massive bleeding or in significant bleeding complicated by coagulopathy (PT or aPTT more than 1.5 times control) [7,157,158]. Patients treated with oral anticoagulants (vitamin K antagonists) present a particular challenge, and thawed FFP is recommended [158] only when prothrombin complex concentrate (PCC) is not available [157]. The most frequently recommended dose is 10 to 15 ml/kg [157,158], but further doses may be required [159].

As with all products derived from human blood, the risks associated with FFP treatment include circulatory overload, ABO incompatibility, transmission of infectious diseases (including the prion diseases), mild allergic reactions, and (particularly) transfusion-related acute lung injury (TRALI) [157,160,161]. FFP and platelet concentrates appear to be the most frequently implicated blood products in TRALI [160-163]. Although the formal link between the administration of FFP, control of bleeding, and an eventual improvement in the outcome of bleeding patients is lacking, most experts would agree that FFP treatment is beneficial in patients with massive bleeding or significant bleeding complicated by coagulopathy.

#### Recommendation 21

We recommend that platelets be administered to maintain a platelet count above 50 × 10^9^/l (grade 1C). We suggest maintenance of a platelet count above 100 × 10^9^/l in patients with multiple trauma who are severely bleeding or have traumatic brain injury (grade 2C). We suggest an initial dose of 4 to 8 platelet concentrates or one aphaeresis pack (grade 2C).

##### Rationale

In medical conditions leading to thrombocytopaenia, haemorrhage does not often occur until the platelet count falls to less than 50 × 10^9^/l, and platelet function decreases exponentially below this point [164-167]. There is no direct evidence to support a particular platelet transfusion threshold in the trauma patient. A consensus development conference sponsored by the National Institutes of Health (NIH) (Bethesda, MD, USA) in 1986 determined that bleeding is unlikely to be caused by thrombocytopaenia at platelet counts of 50 × 10^9^/l or greater and agreed that platelet transfusion is appropriate to prevent or control bleeding associated with deficiencies in platelet number or function [168,169]. The NIH consensus did not consider trauma, but it seems reasonable to recommend that a platelet count of at least 50 × 10^9^/l be maintained following injury.

An argument can be made for maintaining a higher level of platelets, perhaps up to 100 × 10^9^/l, following injury. If a patient has increased fibrin degradation products (for example, in patients with massive bleeding), disseminated intravascular coagulation, or hyperfibrinolysis, this will interfere with platelet function and a higher threshold of 75 × 10^9^/l has been suggested by consensus groups [170,171]. Transfusion threshold levels of up to 100 × 10^9^/l have been suggested for treatment of severe brain injury and massive haemorrhage, but the evidence for the higher threshold is weak [170,171].

When platelet transfusion was introduced in the 1950s, no clinical trials were employed to assess the utility of platelet therapy compared to placebo, and such trials today might be considered unethical. The appropriate dose of platelets is therefore uncertain. Platelet concentrate produced from a unit of whole blood contains 7.5 × 10^10 ^platelets on average and should increase the platelet count by 5 to 10 × 10^9^/l in a 70-kg recipient. Aphaeresis platelet concentrates generally contain approximately 3 to 6 × 10^11 ^platelets, depending on local collection practice, and physicians should be cognizant of the doses provided locally. A pool of 4 to 8 platelet concentrates or a single-donor aphaeresis unit is usually sufficient to provide haemostasis in a thrombocytopaenic, bleeding patient.

If required, the dose of platelets (× 10^9^) can be calculated in more detail from the desired platelet increment, the patient's blood volume in litres (estimated by multiplying the patient's body surface area by 2.5, or 70 ml/kg in an adult), and a correction factor of 0.67 to allow for pooling of approximately 33% of transfused platelets in the spleen.

#### Recommendation 22

We recommend treatment with fibrinogen concentrate or cryoprecipitate if significant bleeding is accompanied by a plasma fibrinogen level of less than 1 g/l. We suggest an initial fibrinogen concentrate dose of 3 to 4 g or 50 mg/kg of cryoprecipitate, which is approximately equivalent to 15 to 20 units in a 70-kg adult. Repeat doses should be guided by laboratory assessment of fibrinogen levels (grade 1C).

##### Rationale

Cryoprecipitate or fibrinogen is used for the correction of both inherited and acquired hypofibrinogenaemia. Their use is based on the assumptions that low fibrinogen levels are associated with a risk of bleeding and that the achievement of higher levels of fibrinogen decreases that risk. The evidence for the clinical efficacy of cryoprecipitate and fibrinogen in trauma patients is limited; no clinical randomised studies have been performed to determine whether the administration of cryoprecipitate or fibrinogen improves clinical outcome in severely bleeding trauma patients. Only indirect observational studies are available, but this evidence suggests that clinically significant bleeding decreases in a variety of clinical scenarios following treatment with both agents. Hypofibrinogenaemia responds well to treatment with cryoprecipitate concentrate [172]. Administration of fibrinogen was associated with bleeding control in patients with generalised, mostly traumatic, bleeding [173]. Administration of 4 g of fibrinogen raised fibrinogen levels from 0.1 to 1 g/l, and bleeding control was achieved in patients with bleeding associated with uterine rupture and abortion [174]. A few observational studies report the successful use of fibrinogen in patients with congenital afibrinogenaemia [175-177]. The optimal initial dose has not been defined, and regional differences in cryoprecipitate and fibrinogen preparations exist, but available evidence suggests that an initial dose of cryoprecipitate or fibrinogen that raises fibrinogen plasma level above 1 g/l will provide sufficient haemostasis [174,176,178].

There are no specific risks related to administration of fibrinogen or cryoprecipitate other than the risks associated with other blood components and the increased risk associated with pooled versus single-donor blood products. Fibrinogen or cryoprecipitate can have unpredictable adverse effects. Of particular concern are allergic reactions and anaphylaxis. There are no reported specific adverse events related to administration of fibrinogen or cryoprecipitate in patients with hypofibrinogenaemia.

### Pharmacological agents

A large body of evidence supports the use of antifibrinolytic agents for the management of bleeding in elective surgery and cardiac surgery patients. For the purpose of these guidelines, we have assumed that these effects are transferable to trauma patients, and our recommendation is based upon this unproven assumption.

#### Recommendation 23

We suggest that antifibrinolytic agents be considered in the treatment of the bleeding trauma patient. Suggested dosages are tranexamic acid (trans-4-aminomethylcyclohexane-1-carboxylic acid) 10 to 15 mg/kg followed by an infusion of 1 to 5 mg/kg per hour, ε-aminocaproic acid 100 to 150 mg/kg followed by 15 mg/kg per hour, or (after a test dose) aprotinin 2 million kallikrein inhibitory units (KIU) immediately followed by 500,000 KIU/hour in an intravenous infusion. Antifibrinolytic therapy should be stopped once bleeding has been adequately controlled (grade 2C).

##### Rationale

Tranexamic acid is a synthetic lysine analogue that is a competitive inhibitor of plasmin and plasminogen. Tranexamic acid is distributed throughout all tissues and the plasma half-life is 120 minutes. There is large variation in the dose employed. *In vitro *studies have suggested that a dose of 10 μg/ml is required to inhibit fibrinolysis [179]. Studies of plasma levels [180] confirmed that the Horrow regimen (10 mg/kg followed by 1 mg/kg per hour) [181], shown to reduce blood loss in cardiac surgery, attained these levels. Other studies have used boluses of up to 5 g per patient with no ill effect [182].

ε-Aminocaproic acid is also a synthetic lysine analogue that has a potency 10-fold weaker than that of tranexamic acid. It is therefore administered in a loading dose of 150 mg/kg followed by a continuous infusion of 15 mg/hour. The initial elimination half-life is 60 to 75 minutes and it must therefore be administered by continuous infusion in order to maintain therapeutic drug levels until the bleeding risk has diminished.

Aprotinin is a broad-spectrum serine protease inhibitor isolated from bovine lung and forms irreversible inhibitory complexes with a number of serine proteases. In particular, it is a powerful antiplasmin agent, and the initial elimination of aprotinin is 1.5 to 2 hours [183]. The 'high-dose' regimen [184] (2 MKIU to patient and cardiopulmonary bypass prime and an infusion of 500,000 KIU/hour) has been shown to reduce perioperative bleeding in open cardiac surgery. However, lower doses do produce adequate antiplasmin effects. A dose of 2 M units is approved for the treatment of hyperfibrinolysis.

The clear efficacy of antifibrinolytic agents in elective surgery and especially in cardiac surgery has been shown in numerous clinical trials [184,185]. A larger number of trials to evaluate the efficacy of aprotinin have been published than assessments of lysine analogue efficacy. It may be possible to extrapolate the benefits of antifibrinolytic agents to bleeding secondary to trauma, but this assumption is not backed by any published data that suggest that the haemostatic response to trauma is similar to the haemostatic response to elective surgery. There is insufficient evidence from RCTs of antifibrinolytic agents in trauma patients to either support or refute a clinically important treatment effect. Further RCTs of antifibrinolytic agents in trauma patients are required [186]. The efficacy of tranexamic acid in trauma will be assessed by the ongoing CRASH (Clinical Randomisation of an Antifibrinolytic in Significant Haemorrhage) II study, in which 20,000 trauma patients worldwide are being randomly assigned to 1 g of tranexamic acid for a period of 10 minutes followed by 1 g infused for a period of 8 hours [187].

The risk of precipitated thrombosis with the use of antifibrinolytic agents has been of major theoretical concern; however, the Cochrane review of antifibrinolytics cites studies that included more than 8,000 patients and demonstrated no increased risk of either arterial or venous thrombotic events [188]. All antifibrinolytics are renally excreted and accumulate in individuals with renal failure, and therefore dosage should be reduced in patients with renal failure. In practice, mild degrees of renal failure do not seem to affect outcome.

Because aprotinin is a bovine protein with an associated risk of anaphylaxis, a test dose must be given. After high-dose aprotinin, as many as 50% of patients develop specific immunoglobulin G antibodies within three months of exposure. The manufacturer (Bayer Pharmaceuticals Corporation, West Haven, CT, USA) estimates a 0.5% overall risk of anaphylactic reactions following aprotinin treatment, which may increase to 6% to 9% following re-exposure [183].

An open study by Mangano and colleagues [189] suggested that aprotinin usage in cardiac surgery was associated with an increased risk of myocardial infarction, stroke, and renal failure. A further publication cited an increased risk of renal problems in patients receiving aprotinin compared to tranexamic acid [190]. Because the study of Mangano and colleagues [189] was open, it remains unclear whether sicker patients in the study may have preferentially received aprotinin. A blinded comparative study of aprotinin versus tranexamic acid versus ε-aminocaproic acid [191] which aims to recruit 3,000 patients will assess safety and efficacy issues in cardiac surgery. At present, in light of the current US Food and Drug Administration warning against the use of aprotinin [192], the greater cost associated with aprotinin use, and the need to give a test dose (often impractical in an emergency situation), we favour the use of tranexamic acid or ε-aminocaproic acid in trauma patients.

### Factor replacement

#### Recommendation 24

We suggest that the use of recombinant activated coagulation factor VII (rFVIIa) be considered if major bleeding in blunt trauma persists despite standard attempts to control bleeding and best-practice use of blood components. We suggest an initial dose of 200 μg/kg followed by two doses of 100 μg/kg administered at 1 and 3 hours following the first dose (grade 2C).

##### Rationale

rFVIIa is not a first-line treatment for bleeding and will be effective only once sources of major bleeding have been controlled. Once major bleeding from damaged vessels has been stopped, rFVIIa may be helpful to induce coagulation in areas of diffuse small vessel coagulopathic bleeding. rFVIIa should be considered only if first-line treatment with a combination of surgical approaches, best-practice use of blood products (RBCs, platelets, FFP, and cryoprecipitate/fibrinogen resulting in Hctabove 24%, plateletsabove 50,000 × 10^9^/l, and fibrinogenabove 0.5 to 1.0 g/l) and correction of severe acidosis, severe hypothermia, and hypocalcaemia (resulting in pHabove 7.20, temperatureabove 32°C, and ionised Ca^++^above 0.8 mmol/l, respectively) fail to control bleeding. Because rFVIIa acts on the patient's own clotting system, a sufficient number of platelets are needed to allow a thrombin burst to be induced by the pharmacological, supraphysiological doses of rFVIIa through direct binding to activated platelets [193,194]. Reduction in platelet count may lead to impaired thrombin generation [195]. Moreover, fibrinogen is required to ensure formation of a stable clot [158,196]. A recent study showed that a pH below 7.20 substantially reduced rFVIIa activity but that a temperature above 32°C only slightly improved rFVIIa activity [197]. Independent of rFVIIa activity, however, pH and body temperature should be restored as near to physiological levels as possible since even small reductions in pH and temperature may result in slower coagulation enzyme kinetics [133,134,198]. Moreover, hypocalcaemia is frequently present in severely injured patients [199] and may require the administration of intravenous calcium with frequent ionised serum calcium measurement [200].

A number of case studies and case series have reported that treatment with rFVIIa can be beneficial in the treatment of coagulopathic bleeding following trauma [201-204]. A recently published multi-centre, randomised, double-blind, placebo-controlled study examined the efficacy of rFVIIa in patients with blunt or penetrating trauma [205]. Patients were randomly assigned to receive either three doses of rFVIIa (200, 100, and 100 μg/kg) or placebo after they had received 6 units of RBCs. The first dose of their assigned medication was administered after transfusion of a further 2 units of RBCs (8 units in total), and a second and third dose were administered 1 and 3 hours later. Treatment with rFVIIa in blunt trauma produced a significant reduction in RBC transfusion requirements and the need for massive transfusions (>20 units of RBCs) in patients with blunt trauma surviving for more than 48 hours and also significantly reduced the incidence of acute respiratory distress syndrome in all patients with blunt trauma. In contrast, no significant effects were observed on RBC transfusion requirements in the penetrating trauma patients in this study, although trends toward reduced RBC requirements and fewer massive transfusions were observed. Therefore, no recommendation to use the drug in this group can be made.

The required dose(s) of rFVIIa is still under debate. Whereas the above dosing recommendation is based on the only published RCT available in trauma patients and is also recommended by a group of European experts [206], Israeli guidelines based on findings from a case series of 36 patients who received rFVIIa on a compassionate-use basis in Israel [201] propose an initial dose of 120 μg/kg (between 100 and 140 μg/kg) and (if required) a second and third dose. Further support for the dose regimen recommended here comes from pharmacokinetic modelling techniques, which have shown that the dose regimen for rFVIIa treatment used in the above-cited RCT is capable of providing adequate plasma levels of drug to support haemostasis [207]. If rFVIIa is administered, the patient's next of kin should be informed that rFVIIa is being used outside the currently approved indications (off-label use), especially since the use of rFVIIa may increase the risk of thromboembolic complications [208].

#### Recommendation 25

We recommend the use of PCC according to the manufacturer's instructions only for the emergency reversal of vitamin K-dependent oral anticoagulants (grade 1C).

##### Rationale

Despite the common use of PCC, there is no clear indication for its use in bleeding non-haemophilia patients. The evidence of clinical efficacy of PCC in patients without haemophilia is limited, and no clinical randomised studies have been performed to determine whether administration of PCC improves clinical outcome in severely bleeding trauma patients. PCC has been used to control bleeding in haemophilia patients [209-211] or to reverse the effect of oral anticoagulant agents [212,213]. The American Society of Anesthesiology recommends the use of PCC in patients with clinical coagulopathy and prolonged PT more than 1.5 times normal [158]. Because there are variations in the production of PCC, the dosage should be determined according to the instructions of the individual manufacturer [214].

Administration of PCC may carry the risk of venous and arterial thrombosis or disseminated intravascular coagulation [215,216]; however, the type of surgery has no influence on the type and severity of these complications [217]. Decreased clearance of activated clotting factor complexes increases the likelihood of these complications in patients with liver disease [218].

#### Recommendation 26

We do not recommend the use of antithrombin III in the treatment of the bleeding trauma patient (grade 1C).

Antithrombin concentrates are indicated in inherited and acquired antithrombin deficiency. Although antithrombin deficiency does occur in consumptive coagulopathy, this is not an isolated condition; all coagulation factors and physiological anticoagulants undergo consumption under these circumstances. The best replacement therapy is FFP. Clinical studies of antithrombin concentrate in severe blunt trauma and in critical care have shown no benefit [219,220].

## Discussion

**Figure 1:**
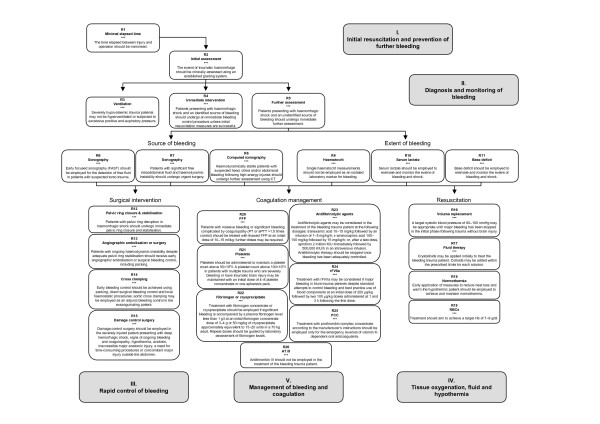
Flowchart of treatment aspects for the bleeding trauma patient which are discussed in this guideline. aPTT, activated partial thromboplastin time; AT III, antithrombin III; CT, computerised tomography; FAST, focused abdominal sonography in trauma; FFP, fresh frozen plasma; Hb, haemoglobin; KIU, kallikrein inhibitory units; PCC, prothrombin complex concentrate; PT, prothrombin time; RBC, red blood cell; rFVIIa, recombinant activated coagulation factor VII.

These guidelines for the management of the bleeding trauma patient are based on a critical appraisal of the published literature and were formulated according to a consensus reached by the author group and the professional societies involved. We have attempted in an evidence-based manner to address a number of critical issues faced by the treating physician confronted with a critically bleeding patient. Figure 1 graphically summarises the recommendations included in this guideline. Unfortunately in emergency medicine, a number of pivotal issues have not been, and due to ethical and practical considerations may never be, addressed in randomised clinical trials. This reality renders the need for best-practice guidelines even more acute.

Although the emphasis in these guidelines has been on the management of critical bleeding in the trauma patient, the high risk of venous thromboembolism in trauma patients should always be kept in mind and thromboprophylactic treatment considered once bleeding has been controlled [221]. In addition, a conscious decision was made to exclude animal studies from the literature reviewed for the development of these guidelines. Because no animal model has accurately mimicked the human coagulation system, RCTs in humans provide the strongest evidence for the management of bleeding in humans.

We have made an effort to consider a number of specific patient subgroups that may require treatment that has been adapted to their physiological condition. There is very little evidence, however, to support specific recommendations for these special patient groups. The physiology of elderly patients with respect to coagulopathy is probably not different than that of younger adults; however, the bleeding patient who has been treated with an anticoagulant or an antiplatelet agent may present with a greater risk of coagulopathic bleeding. We have also considered the management of bleeding following injury in children. A more conservative approach to the surgical management of bleeding in children is generally taken, and there is some evidence that commonly quoted age-related physiological norms are not applicable to the injured child. There is little evidence, however, for specific differences in bleeding and coagulation management in children; therefore, we suggest that these guidelines be applied to both adults and children until research data that are more specific become available.

The apparent weakness of much of the published evidence cited in this work highlights the need for further clinical studies, and underlines the importance of future research that may lead to more clear-cut evidence-based clinical guidelines. The GRADE system employed in developing these guidelines [8] is appropriate in that it allows strong recommendations to be supported by weak clinical evidence in a field in which many of the ideal randomised controlled clinical trials may never be performed. Other systems, such as the grades of recommendation developed by the Oxford Centre for Evidence-Based Medicine [9], may be less dependent on the weight of expert opinion, and therefore the process employed between the published evidence and guideline recommendation must be transparent in order to ensure acceptance and implementation of the guideline. To minimise the bias introduced by individual experts, this guideline employed a nominal group process to develop each recommendation and several Delphi rounds to reach an agreement on the questions to be considered and to reach a final consensus on each recommendation, and the group was composed of a multidisciplinary pan-European group of experts, including the active involvement of representatives from five of the most relevant European professional societies.

## Conclusion

We have made every effort to make these guidelines applicable in daily practice in a variety of clinical settings. We wish to emphasise our conviction that a multidisciplinary approach to the management of the bleeding trauma patient will help create circumstances in which optimal care can be provided. By their very nature, these guidelines reflect the current state-of-the-art and will need to be updated and revised regularly as important new evidence becomes available.

## Key messages

• This guideline to clinical practice provides evidence-based recommendations that were developed by a multidisciplinary task force with respect to many aspects of the acute management of the bleeding trauma patient and that when implemented may lead to improved patient outcomes.

• The time elapsed between injury and operation should be minimised for patients in need of urgent surgical bleeding control, and patients presenting with haemorrhagic shock and an identified source of bleeding should undergo immediate surgical bleeding control unless initial resuscitation measures are successful.

• Patients presenting with haemorrhagic shock and an unidentified source of bleeding should undergo immediate further assessment as appropriate using focused sonography, CT, serum lactate, and/or base deficit measurements.

• A damage control surgical approach is essential in the severely injured patient and may include the closure and stabilisation of pelvic ring disruptions, followed by appropriate angiographic embolisation or surgical bleeding control, including packing.

• This guideline also reviews appropriate physiological targets and suggested use and dosing of blood products, pharmacological agents, and coagulation factor replacement in the bleeding trauma patient.

## Abbreviations

ACS = American College of Surgeons; aPTT = activated partial thromboplastin time; CT = computerised tomography; DPL = diagnostic peritoneal lavage; FAST = focused abdominal sonography in trauma; FFP = fresh frozen plasma; GRADE = Grading of Recommendations Assessment, Development, and Evaluation; Hb = haemoglobin; Hct = haematocrit; ICU = intensive care unit; KIU = kallikrein inhibitory units; MeSH = Medical Subject Heading; MSCT = multi-slice spiral computed tomography; NIH = National Institutes of Health; PCC = prothrombin complex concentrate; PEEP = positive end-expiratory pressure; PT = prothrombin time; RBC = red blood cell; RCT = randomised controlled trial; rFVIIa = recombinant activated coagulation factor VII; TRALI = transfusion-related acute lung injury; TRICC = Transfusion Requirements in Critical Care.

## Competing interests

In the past 5 years, DRS has received honoraria for consulting or lecturing from the following companies: Abbott AG (Baar, Switzerland), Alliance Pharmaceutical Corp. (San Diego, CA, USA), AstraZeneca (London, UK), B. Braun Melsungen AG (Melsungen, Germany), Fresenius Kabi AG (Bad Homburg, Germany), GlaxoSmithKline (Uxbridge, Middlesex, UK), Janssen-Cilag AG (Baar, Switzerland), Novo Nordisk (Bagsvaerd, Denmark), Organon (Roseland, NJ, USA), Roche Pharma (Schweiz) AG (Reinach, Switzerland) and CSLBehring (Marburg, Germany). He serves as chair of the Advanced Bleeding Care (ABC) European medical education initiative and as co-chair of the ABC-Trauma (ABC-T) European medical education initiative, both of which are managed by Thomson Physicians World GmbH (Mannheim, Germany) and supported by educational grants from Novo Nordisk; he represented the European Society of Anaesthesiologists on the ABC-T Task Force. VC is a member of the ABC and ABC-T European medical education initiative faculties. TJC has received honoraria for consulting and lecturing for Novo Nordisk and is a member of the ABC-T European medical education initiative faculty. In the past 5 years, his research group has received research grant funding from Pfizer Inc (New York, NY, USA), the Moulton Foundation (Moulton Charitable Trust, Kent, UK), Novo Nordisk, Barts and the London Special Trustees, Boehringer Ingelheim GmbH (Ingelheim, Germany), and the Anthony Hopkins Memorial Fund. JD is a member of the ABC-T European medical education initiative faculty. EF-M has received honoraria for consulting from PULSION Medical Systems AG (Munich, Germany) and is a member of the ABC-T European medical education initiative faculty. GG is a member of the ABC-T European medical education initiative faculty. PFS has received honoraria for lecturing for Novo Nordisk and is a member of the ABC-T European medical education initiative faculty. BJH has received educational grants or honoraria for lecturing from the following companies: AstraZeneca, GlaxoSmithKline, Novo Nordisk, and sanofi-aventis (Paris, France). RK represented the European Trauma Society on the ABC-T Task Force. EN has received honoraria for consulting or lecturing from the following companies in the past 5 years: Biotest (Frankfurt/Main, Germany), Bristol-Myers Squibb Company (Princeton, NJ, USA), Cook (Cook Biotach Incorporated West Lafayette, IN, USA), Novo Nordisk, Pfizer Inc, and sanofi-aventis. He has received study grants from Bristol-Myers Squibb Company, Choice Medical Communications Ltd. (Hitchin, Hertfordshire, UK), Mundipharma International Limited (Cambridge, Cambridgeshire, UK), and Novo Nordisk. In the past 5 years, YO has received institutional support from Bayer Pharma (France), Novo Nordisk, and LFB (Laboratoire français du fractionnement et des biotechnologies, France) for consulting or lecturing; he represented the European Society of Intensive Care Medicine on the ABC-T Task Force. LR represented the European Society for Emergency Medicine on the ABC-T Task Force. AS represented the European Shock Society on the ABC-T Task Force. J-LV has received honoraria from the following companies: Abbott Laboratories, AM-Pharma (Bunnik, The Netherlands), ArisanPharma Inc. (Framingham, MA, USA), AstraZeneca, Baxter (Deerfield, IL, USA), bioMérieux SA (Lyon, France), Biosite Incorporated (San Diego, CA, USA), Edwards Lifesciences LLC (Irvine, CA, USA), Eli Lilly and Company (Indianapolis, IN, USA), Eisai Inc. (Woodcliff Lake, NJ, USA), Ferring (Saint-Prex, Switzerland), Novo Nordisk, Pfizer Inc, PULSION Medical Systems AG, Takeda Pharmaceutical Company Limited (Osaka, Japan), Theravance, Inc. (South San Francisco, CA, USA), and Wyeth (Madison, NJ, USA). RR has received honoraria for consulting or lecturing from the following companies: Air Liquide (Paris, France), Bayer Pharma Leverkusen, Germany, AGA AB, Linde Gas Therapeutics, Lidingö, Sweden, Eli Lilly and Company, Messer Griesheim (Messer Group GmbH, Sulzbach, Germany), Novo Nordisk, and ZLB Behring; he serves as the chair of the ABC-T European medical education initiative.

## Authors' contributions

All authors participated in the formulation of questions to be addressed in the guideline, screening of abstracts and literature, face-to-face and remote consensus-finding processes, and drafting, review, and revision of the manuscript. All authors read and approved the final manuscript.

## Supplementary Material

Additional file 1 A Word document containing the MeSH terms and limits applied to address guideline literature queries. Click here for file
