# Supplementary material for: Management of bleeding following major trauma: a European guideline
Source: Crit Care. 2007 Feb 13;11(1):R17. doi: 10.1186/cc5686 (PMC2151863; doi:10.1186/cc5686)
Supplement: Additional file 1 — A Word document containing the MeSH terms and limits applied to address guideline literature queries. [file cc5686-S1.doc]

**Additional data file 1.** MeSH terms and limits applied to address guideline literature queries.

| **Question** | | | | **Query** | **Limit** |
| --- | --- | --- | --- | --- | --- |
| **I** | **Initial resuscitation and prevention of further bleeding** | | | | |
|  | **1** | | **Does coagulopathy have an effect on outcome in patients with different types of injury?** | | |
|  |  | | **a** | “Wounds and Injuries”[MeSH] AND “Hemorrhage”[MeSH] AND “Blood coagulation disorders”[MeSH] | Humans |
|  |  | | **b** | ("Emergencies"[MeSH] OR "Emergency Service, Hospital"[MeSH] OR "Emergency Medical Services"[MeSH] OR "Emergency Medical Technicians"[MeSH] OR "Emergency Services, Psychiatric"[MeSH] OR "Emergency Treatment"[MeSH] OR "Emergency Medical Tags"[MeSH] OR "Emergency Nursing"[MeSH] OR "Emergency Medicine"[MeSH] OR "Emergency Medical Service Communication Systems"[MeSH] OR "Ambulances"[MeSH] OR "Air Ambulances"[MeSH] OR "After-Hours Care"[MeSH] OR "Infusions, Intraosseous"[MeSH] OR "Hotlines"[MeSH] OR "Triage"[MeSH] OR "Propranolol"[MeSH] OR "Poison Control Centers"[MeSH] OR "Phentolamine"[MeSH] OR "Nitroprusside"[MeSH] OR "Methylprednisolone Hemisuccinate"[MeSH] OR "Information Systems"[MeSH] OR "Crisis Intervention"[MeSH]) AND ("Wounds and Injuries"[MeSH] OR "injuries"[Subheading] OR "Craniocerebral Trauma"[MeSH] OR "Trauma Severity Indices"[MeSH] OR "Trauma Centers"[MeSH] OR "Cumulative Trauma Disorders"[MeSH] OR "Cerebrovascular Trauma"[MeSH] OR "Multiple Trauma"[MeSH] OR "Trauma, Nervous System"[MeSH] OR "Head Injuries, Penetrating"[MeSH] OR "Abducens Nerve Injury"[MeSH] OR "Optic Nerve Injuries"[MeSH] OR "Coma, Post-Head Injury"[MeSH] OR "Facial Nerve Injuries"[MeSH] OR "Carotid Artery Injuries"[MeSH] OR "Head Injuries, Closed"[MeSH] OR "Spinal Cord Injuries"[MeSH] OR "Traumatology"[MeSH] OR "Brain Injuries"[MeSH]) AND ("Hematologic Diseases"[MeSH] OR "Blood Coagulation Disorders"[MeSH] OR "Blood Coagulation Disorders, Inherited"[MeSH] OR "Disseminated Intravascular Coagulation"[MeSH] OR "Coagulation Protein Disorders"[MeSH] OR "Hemorrhagic Disorders"[MeSH] OR "Factor XII Deficiency"[MeSH]) | Humans |
|  |  | | **c** | “Wounds and Injuries”[MeSH] AND “Blood coagulation disorders”[MeSH] | English, Clinical Trial, Meta-Analysis, Practice Guideline, Randomized Controlled Trial, Review, Humans |
|  | **2** | | **Does control of acid-base balance during the initial resuscitation affect outcome?** | | |
|  |  | | **a** | “Wounds and Injuries”[MeSH] AND “Acid-Base Equilibrium”[MeSH] | Humans |
|  |  | | **b** | "Wounds and Injuries"[MAJR] AND "Acid-Base Equilibrium"[MeSH] | Humans |
|  | **3** | | **Does the degree of initial bleeding affect coagulopathy?** | | |
|  |  | | **a** | “Wounds and Injuries”[MeSH] AND “Hemorrhage”[MeSH] AND “Blood coagulation disorders”[MeSH] AND “Hemostasis”[MeSH] | Humans |
|  |  | | **b** | ("Diagnostic Techniques, Cardiovascular"[MeSH] OR "Hemodynamic Processes"[MeSH] OR "Hemodynamic Phenomena"[MeSH] OR "Blood Circulation"[MeSH]) AND ("Hemorrhage"[MeSH] OR "Brain Stem Hemorrhage, Traumatic"[MeSH] OR "Subarachnoid Hemorrhage"[MeSH] OR "Cerebral Hemorrhage"[MeSH] OR "Cerebral Hemorrhage, Traumatic"[MeSH] OR "Subarachnoid Hemorrhage, Traumatic"[MeSH] OR "Brain Hemorrhage, Traumatic"[MeSH] OR "Intracranial Hemorrhage, Traumatic"[MeSH]) AND ("Hematologic Tests"[MeSH] OR "Hemostasis"[MeSH] OR "Blood Platelet Disorders"[MeSH] OR "Blood Coagulation Disorders"[MeSH]) AND ("Wounds and Injuries"[MeSH] OR "Craniocerebral Trauma"[MeSH] OR "Cerebrovascular Trauma"[MeSH] OR "Multiple Trauma"[MeSH] OR "Trauma, Nervous System"[MeSH] OR "Head Injuries, Penetrating"[MeSH] OR "Carotid Artery Injuries"[MeSH] OR "Head Injuries, Closed"[MeSH] OR "Spinal Cord Injuries"[MeSH] OR "Traumatology"[MeSH] OR "Brain Injuries"[MeSH]) | Humans |
|  |  | | **c** | "Wounds and Injuries"[MeSH] AND "Hemorrhage"[MeSH] AND Hemostasis[MeSH] | Humans |
|  | **4** | | **Does the degree of initial bleeding affect outcome?** | | |
|  |  | | **a** | “Wounds and Injuries”[MeSH] AND “Hemorrhage”[MeSH] AND “Hemostasis”[MeSH] | Humans |
|  | **5** | | **Does initial resuscitation with respect to haemostasis have an effect on outcome in patients with different types of injury?** | | |
|  |  | | **a** | ("Wounds and Injuries"[MeSH] OR "injuries"[Subheading] OR "Craniocerebral Trauma"[MeSH] OR "Trauma Severity Indices"[MeSH] OR "Trauma Centers"[MeSH] OR "Cumulative Trauma Disorders"[MeSH] OR "Cerebrovascular Trauma"[MeSH] OR "Multiple Trauma"[MeSH] OR "Trauma, Nervous System"[MeSH] OR "Head Injuries, Penetrating"[MeSH] OR "Abducens Nerve Injury"[MeSH] OR "Optic Nerve Injuries"[MeSH] OR "Coma, Post-Head Injury"[MeSH] OR "Facial Nerve Injuries"[MeSH] OR "Carotid Artery Injuries"[MeSH] OR "Head Injuries, Closed"[MeSH] OR "Spinal Cord Injuries"[MeSH] OR "Traumatology"[MeSH] OR "Brain Injuries"[MeSH] OR "Emergency Treatment"[MeSH] OR "Emergencies"[MeSH] OR "Critical Care"[MeSH] OR "Emergency Treatment"[MeSH]) AND ("Resuscitation"[MeSH] OR "Resuscitation Orders"[MeSH]) AND ("Hemostatic Techniques"[MeSH] OR "Hemostasis"[MeSH]) | Humans |
|  | **6** | | **Is wound compression effective in preventing bleeding and coagulopathy?** | | |
|  |  | | **a** | ("Emergencies"[MeSH] OR "Emergency Service, Hospital"[MeSH] OR "Emergency Medical Services"[MeSH] OR "Emergency Medical Technicians"[MeSH] OR "Emergency Services, Psychiatric"[MeSH] OR "Emergency Treatment"[MeSH] OR "Emergency Medical Tags"[MeSH] OR "Emergency Nursing"[MeSH] OR "Emergency Medicine"[MeSH] OR "Emergency Medical Service Communication Systems"[MeSH] OR "Ambulances"[MeSH] OR "Air Ambulances"[MeSH] OR "After-Hours Care"[MeSH] OR "Infusions, Intraosseous"[MeSH] OR "Hotlines"[MeSH] OR "Triage"[MeSH] OR "Propranolol"[MeSH] OR "Poison Control Centers"[MeSH] OR "Phentolamine"[MeSH] OR "Nitroprusside"[MeSH] OR "Methylprednisolone Hemisuccinate"[MeSH] OR "Information Systems"[MeSH] OR "Crisis Intervention"[MeSH] OR "Wounds and Injuries"[MeSH] OR "injuries"[Subheading] OR "Craniocerebral Trauma"[MeSH] OR "Trauma Severity Indices"[MeSH] OR "Trauma Centers"[MeSH] OR "Cumulative Trauma Disorders"[MeSH] OR "Cerebrovascular Trauma"[MeSH] OR "Multiple Trauma"[MeSH] OR "Trauma, Nervous System"[MeSH] OR "Head Injuries, Penetrating"[MeSH] OR "Abducens Nerve Injury"[MeSH] OR "Optic Nerve Injuries"[MeSH] OR "Coma, Post-Head Injury"[MeSH] OR "Facial Nerve Injuries"[MeSH] OR "Carotid Artery Injuries"[MeSH] OR "Head Injuries, Closed"[MeSH] OR "Spinal Cord Injuries"[MeSH] OR "Traumatology"[MeSH] OR "Brain Injuries"[MeSH] OR "Emergency Treatment"[MeSH] OR "Emergencies"[MeSH] OR "Critical Care"[MeSH] OR "Emergency Treatment"[MeSH] OR "Hematologic Diseases"[MeSH] OR "Blood Coagulation Disorders"[MeSH] OR "Blood Coagulation Disorders, Inherited"[MeSH] OR "Disseminated Intravascular Coagulation"[MeSH] OR "Coagulation Protein Disorders"[MeSH] OR "Hemorrhagic Disorders"[MeSH] OR "Factor XII Deficiency"[MeSH]) AND "Bandages"[MeSH] AND "Hemorrhage"[MeSH] | Humans |
|  | **7** | | **Does the duration of the pre-hospital phase of initial resuscitation have an effect on outcome in patients with haemorrhagic shock?** | | |
|  |  | | **a** | "Shock, Hemorrhagic"[MeSH] AND ("Emergency Medical Services"[MeSH] OR "Emergencies"[MeSH] OR "Ambulances"[MeSH] OR "Emergency Medical Technicians"[MeSH] OR "Emergency Treatment"[MeSH] OR "Emergency Medical Tags"[MeSH] OR "Emergency Nursing"[MeSH] OR "Emergency Medicine"[MeSH] OR "Emergency Medical Service Communication Systems"[MeSH] OR "Air Ambulances"[MeSH]) AND (pre-hospital OR prehospital) | Humans |
|  | **8** | | **Does the amount of bleeding and type of injury influence the selection of the hospital to which the trauma patient should be transported?** | | |
|  |  | |  | "Health Care Category"[MAJR] AND "Hemorrhage"[MeSH] AND "Wounds and Injuries"[MeSH] AND ("Emergencies"[MeSH] OR "Emergency Treatment"[MeSH] OR "Emergency Medicine"[MeSH] OR "Emergency Medical Technicians"[MeSH]) |  |
| **II** | **Diagnosis and monitoring of bleeding** | | | | |
|  | **1** | **Is there evidence to support a correlation between the mechanism of injury and the risk of bleeding?** | | | |
|  |  | **a** | | “Wounds and Injuries”[MAJR] AND “Hemorrhage”[MeSH] AND correlation[All Fields] | Humans |
|  | **2** | **Which clinical signs are most appropriate to detect the patient who is actively bleeding?** | | | |
|  |  | **a** | | “Wounds and Injuries”[MeSH] AND “Hemorrhage”[MeSH] AND “Signs and Symptoms”[MeSH] AND (“Emergencies”[MeSH] OR "Emergency Treatment"[MeSH] OR "Emergency Medicine"[MeSH] OR "Emergency Medical Technicians"[MeSH]) | Humans |
|  |  | **b** | | "Wounds and Injuries"[MeSH] AND "Hemorrhage"[MeSH] AND ("Emergencies"[MeSH] OR "Emergency Treatment"[MeSH] OR "Emergency Medicine"[MeSH] OR "Emergency Medical Technicians"[MeSH]) AND ("Diagnosis"[MeSH] OR "Signs and Symptoms"[MeSH]) | Humans |
|  | **3** | **Which laboratory parameters (biochemical tests) are most appropriate to detect the patient who is actively bleeding?** | | | |
|  |  | **a** | | “Wounds and Injuries” [MeSH] AND “Hemorrhage” [MeSH] AND (“Clinical Chemistry Tests”[MeSH] OR “Monitoring, Physiologic” [MeSH] OR “Chemistry, Clinical”[MeSH] OR “Biological Markers”[MeSH]) | Humans |
|  |  | **b** | | "Wounds and Injuries" [MeSH] AND "Hemorrhage" [MeSH] AND ("Clinical Chemistry Tests"[MeSH] OR "Monitoring, Physiologic" [MeSH] OR "Chemistry, Clinical"[MeSH] OR "Biological Markers"[MeSH] OR "Intercellular Signaling Peptides and Proteins"[MeSH]) | Humans |
|  | **4** | **Which imaging diagnostic tools are most appropriate to detect the patient who is actively bleeding?** | | | |
|  |  | **a** | | "wounds and injuries"[MeSH] AND "Hemorrhage"[MeSH] AND "Diagnostic Imaging"[MeSH] AND ("Emergencies"[MeSH] OR "Emergency Treatment"[MeSH] OR "Emergency Medicine"[MeSH] OR "Emergency Medical Technicians"[MeSH]) | Humans |
|  | **5** | **Is there evidence to support the use of a specific score to assess the axtent of bleeding?** | | | |
|  |  | **a** | | "Hemorrhage"[MeSH] AND "Trauma Severity Indices"[MeSH] AND "Wounds and Injuries"[MeSH] | humans, 5 years |
| **III** | **Rapid control of bleeding** | | | | |
|  | **1** | **Can the mechanism of injury (e.g. blunt vs. penetrating trauma) be used as a determinant for deciding which patients in haemorrhagic shock are candidates for surgical bleeding control?** | | | |
|  |  | **a** | | “Wounds and Injuries”[MAJR] AND “Hemorrhage”[MeSH] AND “Shock, Hemorrhagic”[MeSH] AND (“Emergencies[MeSH] OR “Emergency Treatment”[MeSH] OR “Emergency Medicine”[MeSH] OR “Emergency Medical Technicians”[MeSH]) | Humans |
|  | **2** | **Does angiographic embolisation improve the outcome of patients with haemorrhagic shock and pelvic ring disruption?** | | | |
|  |  | **a** | | “Embolization, Therapeutic”[MeSH] AND (“Shock, Hemorrhagic”[MeSH] OR “Fractures”[MeSH]) | Humans |
|  | **3** | **What are the characteristics of patients with free intraabdominal fluid according to FAST in whom secondary diagnostics (i.e. CT scan) can be safely performed?** | | | |
|  |  | **a** | | “Wounds and Injuries”[MeSH] AND (“Diagnostic Imaging”[MeSH] OR “Ultrasonography”[MeSH]) AND “Ascitic Fluid”[MeSH] | Humans |
|  | **4** | **What characterises the patient in heamorrhagic shock in whom in whom immediate aortic cross-clamping is warranted?** | | | |
|  |  | **a** | | “Wounds and Injuries”[MeSH] AND (“Diagnostic Imaging”[MeSH] OR “Ultrasonography”[MeSH]) AND “Ascitic Fluid”[MeSH] “Shock, Hemorrhagic”[MeSH] AND (“Angioscopy”[MeSH] OR “Heart, Assist Devices”[MeSH] OR “Cardiovascular Surgical Procedures”[MeSH] OR “Surgical Procedures Minimally Invasive”[MeSH]) | Humans |
|  | **5** | **Does the elapsed time from admission to OR influence outcome for trauma patients who need emergency surgery?** | | | |
|  |  | **a** | | ("Emergency Medical Services"[MeSH] OR "Emergencies"[MeSH] OR "Emergency Treatment"[MeSH] OR "Critical Care"[MeSH]) AND "Hemorrhage"[MeSH] AND "Outcome and Process Assessment (Health Care)"[MeSH] AND ("Wounds and Injuries"[MeSH] OR "injuries"[Subheading] OR "Craniocerebral Trauma"[MeSH] OR "Trauma Severity Indices"[MeSH] OR "Trauma Centers"[MeSH] OR "Cumulative Trauma Disorders"[MeSH] OR "Cerebrovascular Trauma"[MeSH] OR "Multiple Trauma"[MeSH] OR "Trauma, Nervous System"[MeSH] OR "Head Injuries, Penetrating"[MeSH] OR "Abducens Nerve Injury"[MeSH] OR "Optic Nerve Injuries"[MeSH] OR "Coma, Post-Head Injury"[MeSH] OR "Facial Nerve Injuries"[MeSH] OR "Carotid Artery Injuries"[MeSH] OR "Head Injuries, Closed"[MeSH] OR "Spinal Cord Injuries"[MeSH] OR "Traumatology"[MeSH] OR "Brain Injuries"[MeSH]) | Humans |
|  | **6** | **Does urgent surgery to control haemorrhage improve the outcome of patients with haemorrhagic shock and pelvic ring disruption?** | | | |
|  |  | **a** | | "Hip Fractures"[MeSH] AND "Shock, Hemorrhagic"[MeSH] | Humans |
|  | **7** | **What characterises the patient with free intraabdominal fluid according to FAST who requires immediate laparotomy?** | | | |
|  |  | **a** | | "Ascitic Fluid"[MeSH] AND "Laparotomy"[MeSH] AND ("Wounds and Injuries"[MeSH] OR "injuries"[Subheading] OR "Craniocerebral Trauma"[MeSH] OR "Trauma Severity Indices"[MeSH] OR "Trauma Centers"[MeSH] OR "Cumulative Trauma Disorders"[MeSH] OR "Cerebrovascular Trauma"[MeSH] OR "Multiple Trauma"[MeSH] OR "Trauma, Nervous System"[MeSH] OR "Head Injuries, Penetrating"[MeSH] OR "Abducens Nerve Injury"[MeSH] OR "Optic Nerve Injuries"[MeSH] OR "Coma, Post-Head Injury"[MeSH] OR "Facial Nerve Injuries"[MeSH] OR "Carotid Artery Injuries"[MeSH] OR "Head Injuries, Closed"[MeSH] OR "Spinal Cord Injuries"[MeSH] OR "Traumatology"[MeSH] OR "Brain Injuries"[MeSH]) | Humans |
| **IV** | **Tissue oxygenation, type of fluid and hypothermia** | | | | |
|  | **1** | **What is the corridor for the haematocrit to be achieved for adequate tissue oxygenation?** | | | |
|  |  | **a** | | "Hematocrit"[MeSH] AND ("Oxygen Consumption"[MeSH] OR "Blood Gas Monitoring, Transcutaneous"[MeSH] OR "Blood Gas Analysis"[MeSH] OR "Gases"[MeSH] OR "Cell Respiration"[MeSH] OR "Blood Substitutes"[MeSH] OR "Blood Chemical Analysis"[MeSH] OR "Respiratory Function Tests"[MeSH] OR "Oximetry"[MeSH] OR "Blood Pressure"[MeSH] OR "Venous Pressure"[MeSH] OR "Hypotension"[MeSH]) AND ("Emergencies"[MeSH] OR "Emergency Treatment"[MeSH] OR "Emergency Medicine"[MeSH] OR "Emergency Medical Technicians"[MeSH] OR "Wounds and Injuries"[MeSH] OR "Craniocerebral Trauma"[MeSH] OR "Cerebrovascular Trauma"[MeSH] OR "Multiple Trauma"[MeSH] OR "Trauma, Nervous System"[MeSH] OR "Head Injuries, Penetrating"[MeSH] OR "Carotid Artery Injuries"[MeSH] OR "Head Injuries, Closed"[MeSH] OR "Spinal Cord Injuries"[MeSH] OR "Traumatology"[MeSH] OR "Brain Injuries"[MeSH]) | Humans |
|  | **2** | **How should volume loading be managed?** | | | |
|  |  |  | | ("Blood Substitutes"[MeSH] OR "Fluorocarbons"[MeSH] OR "Plasma Substitutes"[MeSH] OR "Fluorocarbons"[MeSH] OR "Plasma Substitutes"[MeSH] OR "Fluid Therapy"[MeSH] OR "Rehydration Solutions"[MeSH] OR "Solutions"[MeSH] OR "Colloids"[MeSH] OR "Sodium Chloride"[MeSH] OR "Saline Solution, Hypertonic"[MeSH]) AND ("Wounds and Injuries"[MeSH] OR "Craniocerebral Trauma"[MeSH] OR "Cerebrovascular Trauma"[MeSH] OR "Multiple Trauma"[MeSH] OR "Trauma, Nervous System"[MeSH] OR "Head Injuries, Penetrating"[MeSH] OR "Carotid Artery Injuries"[MeSH] OR "Head Injuries, Closed"[MeSH] OR "Spinal Cord Injuries"[MeSH] OR "Traumatology"[MeSH] OR "Brain Injuries"[MeSH]) AND ("Infusions, Parenteral"[MeSH] OR "Infusions, Intravenous"[MeSH]) | Humans |
|  | **3** | **Does the blood pressure achived during initial resuscitation influence morbidity or outcome in the trauma patient?** | | | |
|  |  |  | | ("Blood Pressure Determination"[MeSH] OR "Blood Pressure"[MeSH] OR "Blood Pressure Monitoring, Ambulatory"[MeSH] OR "Hypertension"[MeSH] OR "Venous Pressure"[MeSH] OR "Hypotension"[MeSH]) AND ("Wounds and Injuries"[MeSH] OR "Craniocerebral Trauma"[MeSH] OR "Cerebrovascular Trauma"[MeSH] OR "Multiple Trauma"[MeSH] OR "Trauma, Nervous System"[MeSH] OR "Head Injuries, Penetrating"[MeSH] OR "Carotid Artery Injuries"[MeSH] OR "Head Injuries, Closed"[MeSH] OR "Spinal Cord Injuries"[MeSH] OR "Traumatology"[MeSH] OR "Brain Injuries"[MeSH]) AND ("Emergencies"[MeSh] OR "Emergency Treatment"[MeSH] OR "Emergency Medicine"[MeSh] OR "Emergency Medical Technicians"[MeSH]) | Humans, 25 years |
|  | **4** | **Does the type of fluid used for initial resuscitation influence morbidity or outcome in the trauma patient?** | | | |
|  |  |  | | ("Blood Substitutes"[MeSH] OR "Fluorocarbons"[MeSH] OR "Plasma Substitutes"[MeSH] OR "Fluorocarbons"[MeSH] OR "Plasma Substitutes"[MeSH] OR "Fluid Therapy"[MeSH] OR "Rehydration Solutions"[MeSH] OR "Solutions"[MeSH] OR "Colloids"[MeSH] OR "Sodium Chloride"[MeSH] OR "Saline Solution, Hypertonic"[MeSH]) AND ("Wounds and Injuries"[MeSH] OR "Craniocerebral Trauma"[MeSH] OR "Cerebrovascular Trauma"[MeSH] OR "Multiple Trauma"[MeSH] OR "Trauma, Nervous System"[MeSH] OR "Head Injuries, Penetrating"[MeSH] OR "Carotid Artery Injuries"[MeSH] OR "Head Injuries, Closed"[MeSH] OR "Spinal Cord Injuries"[MeSH] OR "Traumatology"[MeSH] OR "Brain Injuries"[MeSH]) AND ("Emergencies"[MeSH] OR "Emergency Treatment"[MeSH] OR "Emergency Medicine"[MeSH] OR "Emergency Medical Technicians"[MeSH]) | Humans |
|  | **5** | **Does controlled mild hypothermia (34°C) affect outcome or morbidity in the trauma patient compared to normothermia?** | | | |
|  |  |  | | ("Hypothermia"[MeSH] OR "Gastric Hypothermia"[MeSH] OR "Hypothermia, Induced"[MeSH] OR "Circulatory Arrest, Deep Hypothermia Induced"[MeSH]) AND "Outcome and Process Assessment (Health Care)"[MeSH] AND ("Wounds and Injuries"[MeSH] OR "injuries"[Subheading] OR "Craniocerebral Trauma"[MeSH] OR "Trauma Severity Indices"[MeSH] OR "Trauma Centers"[MeSH] OR "Cumulative Trauma Disorders"[MeSH] OR "Cerebrovascular Trauma"[MeSH] OR "Multiple Trauma"[MeSH] OR "Trauma, Nervous System"[MeSH] OR "Head Injuries, Penetrating"[MeSH] OR "Abducens Nerve Injury"[MeSH] OR "Optic Nerve Injuries"[MeSH] OR "Coma, Post-Head Injury"[MeSH] OR "Facial Nerve Injuries"[MeSH] OR "Carotid Artery Injuries"[MeSH] OR "Head Injuries, Closed"[MeSH] OR "Spinal Cord Injuries"[MeSH] OR "Traumatology"[MeSH] OR "Brain Injuries"[MeSH]) | Humans |
| **V** | **Management of bleeding and coagulation** | | | | |
|  | **1-5** | | **Pharmacological agents to support coagulation** | | |
|  |  | | **a** | "Antifibrinolytic Agents"[MeSH] AND ("Hemorrhage"[MeSH] OR "Brain Stem Hemorrhage, Traumatic"[MeSH] OR "Subarachnoid Hemorrhage"[MeSH] OR "Cerebral Hemorrhage"[MeSH] OR "Cerebral Hemorrhage, Traumatic"[MeSH] OR "Subarachnoid Hemorrhage, Traumatic"[MeSH] OR "Brain Hemorrhage, Traumatic"[MeSH] OR "Intracranial Hemorrhage, Traumatic"[MeSH]) | Humans |
|  |  | | **b** | "Antifibrinolytic Agents"[MeSH] AND ("Hemorrhage"[MeSH] OR "Brain Stem Hemorrhage, Traumatic"[MeSH] OR "Subarachnoid Hemorrhage"[MeSH] OR "Cerebral Hemorrhage"[MeSH] OR "Cerebral Hemorrhage, Traumatic"[MeSH] OR "Subarachnoid Hemorrhage, Traumatic"[MeSH] OR "Brain Hemorrhage, Traumatic"[MeSH] OR "Intracranial Hemorrhage, Traumatic"[MeSH]) | Humans |
|  | **6-9** | | **Platelets** | | |
|  |  | | **a** | ("Blood Platelets"[MeSH] OR "Thrombocytopenia"[MeSH]) AND ("Wounds and Injuries"[MeSH] OR "Craniocerebral Trauma"[MeSH] OR "Cerebrovascular Trauma"[MeSH] OR "Multiple Trauma"[MeSH] OR "Trauma, Nervous System"[MeSH] OR "Head Injuries, Penetrating"[MeSH] OR "Carotid Artery Injuries"[MeSH] OR "Head Injuries, Closed"[MeSH] OR "Spinal Cord Injuries"[MeSH] OR "Traumatology"[MeSH] OR "Brain Injuries"[MeSH]) | Humans, 2005-1980 |
|  |  | | **b** | ("Blood Platelets"[MeSH] OR "Thrombocytopenia"[MeSH]) AND ("Wounds and Injuries"[MeSH] OR "Craniocerebral Trauma"[MeSH] OR "Cerebrovascular Trauma"[MeSH] OR "Multiple Trauma"[MeSH] OR "Trauma, Nervous System"[MeSH] OR "Head Injuries, Penetrating"[MeSH] OR "Carotid Artery Injuries"[MeSH] OR "Head Injuries, Closed"[MeSH] OR "Spinal Cord Injuries"[MeSH] OR "Traumatology"[MeSH] OR "Brain Injuries"[MeSH]) | Humans, 1979-end |
|  | **11-12** | | **Red Cells** | | |
|  |  | | **a** | ("Erythrocytes"[MeSH] OR "Erythrocyte Transfusion"[MeSH]) AND ("Wounds and Injuries"[MeSH] OR "Craniocerebral Trauma"[MeSH] OR "Cerebrovascular Trauma"[MeSH] OR "Multiple Trauma"[MeSH] OR "Trauma, Nervous System"[MeSH] OR "Head Injuries, Penetrating"[MeSH] OR "Carotid Artery Injuries"[MeSH] OR "Head Injuries, Closed"[MeSH] OR "Spinal Cord Injuries"[MeSH] OR "Traumatology"[MeSH] OR "Brain Injuries"[MeSH]) | Humans, 2005-1980 |
|  |  | | **b** | ("Erythrocytes"[MeSH] OR "Erythrocyte Transfusion"[MeSH]) AND ("Wounds and Injuries"[MeSH] OR "Craniocerebral Trauma"[MeSH] OR "Cerebrovascular Trauma"[MeSH] OR "Multiple Trauma"[MeSH] OR "Trauma, Nervous System"[MeSH] OR "Head Injuries, Penetrating"[MeSH] OR "Carotid Artery Injuries"[MeSH] OR "Head Injuries, Closed"[MeSH] OR "Spinal Cord Injuries"[MeSH] OR "Traumatology"[MeSH] OR "Brain Injuries"[MeSH]) | Humans, 1980-end |
|  | **13-17** | | **FFP** | | |
|  |  | | **a** | ("Plasma"[MeSH] OR "Plasma Exchange"[MeSH]) AND ("Wounds and Injuries"[MeSH] OR "Craniocerebral Trauma"[MeSH] OR "Cerebrovascular Trauma"[MeSH] OR "Multiple Trauma"[MeSH] OR "Trauma, Nervous System"[MeSH] OR "Head Injuries, Penetrating"[MeSH] OR "Carotid Artery Injuries"[MeSH] OR "Head Injuries, Closed"[MeSH] OR "Spinal Cord Injuries"[MeSH] OR "Traumatology"[MeSH] OR "Brain Injuries"[MeSH]) | Humans |
|  | **18-21** | | **Fibrinogen and Cryoprecipitate** | | |
|  |  | | **a** | ("Fibrinogen"[MeSH] OR "cryoprecipitate coagulum"[Substance Name] OR "Nour-Eldin fraction"[Substance Name]) AND ("Wounds and Injuries"[MeSH] OR "Craniocerebral Trauma"[MeSH] OR "Cerebrovascular Trauma"[MeSH] OR "Multiple Trauma"[MeSH] OR "Trauma, Nervous System"[MeSH] OR "Head Injuries, Penetrating"[MeSH] OR "Carotid Artery Injuries"[MeSH] OR "Head Injuries, Closed"[MeSH] OR "Spinal Cord Injuries"[MeSH] OR "Traumatology"[MeSH] OR "Brain Injuries"[MeSH]) | Humans |
|  | **22-26** | | **Prothrombin complex concentrates** | | |
|  |  | | **a** | ("Thromboplastin"[MeSH] OR "Partial Thromboplastin Time"[MeSH] OR "Factor XI"[MeSH] OR "thromboplastin apoprotein, human"[Substance Name] OR "prothrombinase complex"[Substance Name] OR "Factor IXa"[MeSH] OR "Prothrombin Time"[MeSH] OR "Factor VII"[MeSH] OR "Factor IX"[MeSH] OR "prothrombin complex concentrates"[Substance Name]) AND ("Wounds and Injuries"[MeSH] OR "Craniocerebral Trauma"[MeSH] OR "Cerebrovascular Trauma"[MeSH] OR "Multiple Trauma"[MeSH] OR "Trauma, Nervous System"[MeSH] OR "Head Injuries, Penetrating"[MeSH] OR "Carotid Artery Injuries"[MeSH] OR "Head Injuries, Closed"[MeSH] OR "Spinal Cord Injuries"[MeSH] OR "Traumatology"[MeSH] OR "Brain Injuries"[MeSH]) | Humans |
|  |  | |  |  |  |
